# Prevalence and Predictors of Persistent Speech Sound Disorder at Eight Years Old: Findings From a Population Cohort Study

**DOI:** 10.1044/2015_JSLHR-S-14-0282

**Published:** 2016-08

**Authors:** Yvonne Wren, Laura L. Miller, Tim J. Peters, Alan Emond, Sue Roulstone

**Affiliations:** a Bristol Speech and Language Therapy Research Unit, North Bristol NHS Trust, Bristol, United Kingdom; b School of Oral and Dental Sciences, University of Bristol, United Kingdom; c School of Social and Community Medicine, University of Bristol, United Kingdom; d School of Clinical Sciences, University of Bristol, United Kingdom; e Centre for Child and Adolescent Health, School of Social and Community Medicine, University of Bristol, United Kingdom; f Faculty of Health and Life Sciences, University of the West of England, Bristol, United Kingdom

## Abstract

**Purpose:**

The purpose of this study was to determine prevalence and predictors of persistent speech sound disorder (SSD) in children aged 8 years after disregarding children presenting solely with common clinical distortions (i.e., residual errors).

**Method:**

Data from the Avon Longitudinal Study of Parents and Children (Boyd et al., 2012) were used. Children were classified as having persistent SSD on the basis of percentage of consonants correct measures from connected speech samples. Multivariable logistic regression analyses were performed to identify predictors.

**Results:**

The estimated prevalence of persistent SSD was 3.6%. Children with persistent SSD were more likely to be boys and from families who were not homeowners. Early childhood predictors identified as important were weak sucking at 4 weeks, not often combining words at 24 months, limited use of word morphology at 38 months, and being unintelligible to strangers at age 38 months. School-age predictors identified as important were maternal report of difficulty pronouncing certain sounds and hearing impairment at age 7 years, tympanostomy tube insertion at any age up to 8 years, and a history of suspected coordination problems. The contribution of these findings to our understanding of risk factors for persistent SSD and the nature of the condition is considered.

**Conclusion:**

Variables identified as predictive of persistent SSD suggest that factors across motor, cognitive, and linguistic processes may place a child at risk.

Despite variation in the rate of speech development, most children who are native speakers of English master accurate production of all vowels and consonants by age 8 years (Dodd, Hulm, Hua, & Crosbie, 2003; James, 2001; Smit, 1993a, 1993b; Templin, 1957). However, some individuals experience difficulties with speech production beyond this age and even into adulthood (Bralley & Stoudt, 1977; Felsenfeld, Broen, & McGue, 1992). These children with persistent speech sound disorder (SSD) constitute a substantial proportion (8.8%) of clinical caseloads (Broomfield & Dodd, 2004). This article focuses on those children with clinically significant and persistent SSD that goes beyond the /s/ and /r/ distortions defined by Shriberg (1993) as common clinical distortions. Using data from a large longitudinal population study, prevalence at age 8 years and associated risk factors are identified to aid our understanding of persistent SSD in the clinical setting.

## Previous Studies of Prevalence of SSD

Studies of the prevalence of SSD have reported rates ranging from 2.3% to 24.6% (Eadie et al., 2015; Jessup, Ward, Cahill, & Heating, 2008; Keating, Turrell, & Ozanne, 2001; Law, Boyle, Harris, Harkness, & Nye, 2000; McKinnon, McLeod, & Reilly, 2007; Shriberg, Austin, Lewis, McSweeny, & Wilson, 1997b; Shriberg, Tomblin, & McSweeny, 1999). This variation is most likely explained by two methodological issues. First, there have been differences in the sampling process used. For example, decreasing prevalence rates have been associated with increasing age (McKinnon et al., 2007; Shriberg et al., 1997b), and differences in inclusion criteria relating to speech only versus speech and language impairment (Jessup et al., 2008) and variations in the definition of SSD in terms of which types of errors constitute the disorder (Shriberg et al., 1999) may all affect the final estimated figure. Second, studies have used a variety of methods to identify SSD, including parent or teacher identification (Keating et al., 2001; McKinnon et al., 2007), formal assessments (Eadie et al., 2015; Jessup et al., 2008), and speech sampling (Shriberg et al., 1999). The variability in methodology and dearth of age-specific prevalence figures make it difficult to draw firm conclusions about the prevalence of persistent SSD. Therefore, there is a need for an estimate to be determined from population-based data using a robust means of case identification.

## Factors Associated With Persistent SSD

Understanding the risk factors associated with persistent SSD may provide important clues regarding the nature of the disorder. In order to develop a model of risk factors that might form the basis of a new investigation, studies that investigated factors associated with SSD in early childhood and at school age were examined to identify putative factors. Risk factors that occur early in a child's life do not necessarily play a causative role; however, they may enable us to predict which children are likely to go on to have the more resistant and persistent disorders and thus facilitate early identification and prioritization for intervention. Furthermore, the identification of early risk factors may indicate causative mechanisms that are in themselves amenable to interventions. Factors identified during school age are associated with a concurrent diagnosis of SSD and therefore cannot be considered risk factors. Nevertheless, they may suggest candidate variables that could be investigated at earlier ages.

Tables 1 and 2 summarize studies that have focused on factors in early childhood and school age that are associated specifically with SSD. Examination of the factors studied shows no consistent modeling of risk for SSD across studies, and thus the factors investigated vary in each study. The different research designs and sampling processes further undermine the comparability of findings and thus the possibility of drawing firm conclusions about which factors are predictive of SSD.

**Table 1.** Summary of studies of early childhood risk factors associated with speech sound disorder (SSD).

Study	Country	Research design	Sample size	Sample age at recruitment (years;months)	Demographic factors				Family and environmental factors									Early development							
					Male gender	Ethnicity	Socioeconomic status	Parental marital status	Bilingualism	Family history	Family size	Multiple births	Pre- and perinatal factors	Maternal age	Maternal mental health	Maternal vocabulary	Birth order	Birth weight	Use of pacifiers	Delay in vocalizations and early language	Delay in gross motor skills	Difficulties with feeding and dribbling	General delay	Medical conditions/health	History of hearing/ear, nose, and throat problems
Campbell et al. (2003)	United States	Case/control: longitudinal	100 SSD, 539 controls	3;0–3;2	Y	N	Y^a^			Y															N
Delgado et al. (2005)	United States	Ecological	6,835 SSD, 946,177 controls	All pre-school age			N^b^	N^b^				Y	Y/N^c^											Y	
Eadie et al. (2015)	Australia	Population cohort	1,494 normative sample	4;0–4;8	Y		Y			Y		N	N	N	N	Y	N	N		Y	Y				
Fox et al. (2002)	Germany	Case/control: cross-sectional	65 SSD, 48 controls	2;7–7;2	N					Y			Y						Y						N
Broomfield & Dodd (2004) ^d^	United Kingdom	Cohort	320 SSD of different subtypes	0;0–11;11+	Y		N		N	N	N								N	N		N	Y	N	
Felsenfeld & Plomin (1997)	United States	Case/control	66 at risk for SSD, 90 classified as low risk	7;0–7;11	N					Y															
Highman et al. (2008)	Australia	Case/control	20 childhood apraxia of speech, 20 controls	3;1–5;0																Y	Y	Y			
Lewis & Freebairn (1997)	United States	Case/control	34 with affected relative, 25 with no affected relatives	3;0–8;8						Y															
Wolke & Meyer (1999)	Germany	Population cohort (data analyzed as case/control)	264 preterm, 264 matched-term controls	6;3									Y												

*Note.* Y = significant relationship was observed; N = no significant relationship was observed. Blank boxes indicate that the aspect was not investigated.

a Two proxies for SES were used: maternal education and Medicaid health insurance categories. In the final multivariate logistic regression, maternal education remained important and Medicaid was no longer a significant risk.

b Showed a decreased risk for SSD (i.e., was protective).

c Maternal alcohol use, maternal age over 35 years, and maternal medical history factors were associated with an increased risk for SSD. Low birth weight, preterm delivery, maternal tobacco use during pregnancy, and presence of a labor or pregnancy complication showed a neither increased nor decreased risk for SSD. Low Apgar scores and maternal age younger than 18 years were associated with a decreased risk for SSD.

d Results were not consistent for all subtypes (articulation, phonological delay, consistent deviant, inconsistent deviant).

**Table 2.** Summary of studies of school-age factors associated with speech sound disorder.

Study	Country	Research design	Sample size	Sample age at recruitment (years;months)	Demographic factor: male gender	Family and environment factor: family history	Later development		
							Language impairment	Literacy difficulties	Hearing loss and otitis media with effusion
Bishop & Adams (1990)	United Kingdom	Longitudinal cohort	83	8;6			Y	Y	
Broomfield & Dodd (2004)	United Kingdom	Ecological	320	0;0–11;11+			Y		
Lewis et al. (2006)	United States	Longitudinal cohort	38	3;0–7;0	Y	Y	Y		
Raitano et al. (2004)	United States	Case/control	142	5;0–6;11			Y		
Shriberg et al. (1999)	United States	Cohort	1,328	6;0–6;11			Y		
Bird et al. (1995)	United Kingdom	Case/control	31	5;0–7;4	Y			Y	
Felsenfeld et al. (1994)	United States	Longitudinal cohort	52	32;0–34;11				Y	
Gillon & Moriarty (2007)	New Zealand	Single case	3	6;3, 6;10, 7;10				Y	
Hesketh (2004)	United Kingdom	Longitudinal cohort	35	6;6–7;6				Y	
Larrivee & Catts (1999)	United States	Case/control	57	5;8–7;3				Y	
Leitao et al. (2000)	Australia	Longitudinal cohort	21	5;4–6;2				Y	
Nathan et al. (2004)	United Kingdom	Case/control	39	4;0–5;11				Y	
Peterson et al. (2009)	United States	Case/control	124	7;0–9;11				Y	
Rvachew (2007)	Canada	Case/control	68	4;0–5;11				Y	
Sutherland & Gillon (2005, 2007)	New Zealand	Case/control	9	3;9–5;3				Y	
Paradise et al. (2005, 2007)	United States	Longitudinal cohort	391	0;0–11;11+					N

*Note.* Y = significant relationship was observed; N = no significant relationship was observed. Blank boxes indicate that the aspect was not investigated.

An additional category of studies that have used a broad classification of speech-language impairment was considered to see whether this achieved greater clarity. This produced a number of additional candidate variables, which are summarized in Table 3 and can be considered alongside the findings of the early childhood and school-age risk factor studies. When all the literature is considered together, a pattern of putative risk factors begins to emerge in terms of the child's demography; family and environmental context; and developmental progression in speech and language, literacy, learning, and other general development.

**Table 3.** Summary of studies of risk factors associated with speech and language impairment.

Study	Country	Research design	Sample size	Sample age at recruitment (years;months)	Demographic factors			Family and environmental factors						Early development factors			
					Gender	Parental literacy levels	Socioeconomic status	Bilingualism	Family history	Family size/birth order	Overcrowding	Absence of preschool education	Pre- and perinatal factors	Low birth weight	Delay in early language	Delay in motor skills	Hearing/ear, nose, and throat problems
Choudhury & Benasich (2003)	United States	Longitudinal cohort	136	<0;6–3;0					Y								
Harrison & McLeod (2010)	Australia	Longitudinal cohort	4,983	4;3–5;7	Y			N		Y			Y				Y
Reilly et al. (2007)	Australia	Longitudinal cohort	1,720	0;8–2;0	Y		Y	Y	Y	Y			Y	Y			
Stanton-Chapman et al. (2002)	United States	Ecological	244,619	6;0–7;11			Y	N		Y			Y/N	Y			
Tomblin et al. (1991)	United States	Longitudinal cohort	662	2;6–5;0	Y		N		Y	Y				N			
Zubrick et al. (2007)	Australia	Case/control	1,766	2;0						Y							
Law et al. (2009)	United Kingdom	Longitudinal cohort	17,176	5;0							Y	Y					
Yliherva et al. (2001)	Finland	Longitudinal cohort	9,322	8;0–8;11										Y			
Bishop & Edmundson (1987)	United Kingdom	Longitudinal cohort	87	4;0, 4;6, 5;6											Y		
Dale et al. (2003)	United Kingdom	Longitudinal cohort	8,386	2;0			N								Y		N
Glogowska et al. (2006)	United Kingdom	Case/control	196	7;0–10;11											Y		
Rescorla (2002)	United States	Case/control	59	6;0–9;11											Y		
Rice et al. (2008)	Australia	Case/control	237	7;0–7;11											Y		
Roulstone et al. (2009)	United Kingdom	Longitudinal cohort	741	2;1, 5;0, 8;0–8;11											Y		
Hill & Bishop (1998)	United Kingdom	Case/control	75	7;0–11;11												Y	
Visscher et al. (2010)	Netherlands	Case/control	210	6;0–9;11												Y	
Visscher et al. (2007)	Netherlands	Case/control	125	6;0–9;11												Y	
Robinson (1991)	United Kingdom	Cohort	82	School age									Y			Y	
Webster et al. (2005)	Canada	Longitudinal cohort	43	6;0–7;11												Y	

*Note.* Y = significant relationship was observed; N = no significant relationship was observed. Blank boxes indicate that the aspect was not investigated.

### Demographic Factors

Demographic factors considered in the studies include the child's gender, ethnicity, socioeconomic status (SES), and parental marital status. An association between male gender and SSD was found in some studies (Broomfield & Dodd, 2004; Campbell et al., 2003; Eadie et al., 2015) but not others (Felsenfeld & Plomin, 1997; Fox, Dodd, & Howard, 2002). Likewise, SES was associated with SSD in some studies (Campbell et al., 2003; Eadie et al., 2015; Law, Rush, Schoon, & Parsons, 2009) but not observed in others (Broomfield & Dodd, 2004) and was even shown in one study to be protective (Delgado, Vagi, & Scott, 2005). Variations in this factor can be influenced by how it is measured. A range of methods were used in these studies, including maternal education, health insurance category, and parental literacy levels.

### Family and Environmental Factors

Family and environment factors covered a wide range of areas, including family history of SSD, birth order and family size, multiple births, bilingualism in the home, overcrowding, and preschool education. With regard to family history, Campbell et al. (2003), Eadie et al. (2015); Fox et al. (2002), Felsenfeld and Plomin (1997), and Lewis and Freebairn (1997) all showed a positive association with SSD, though this was not replicated by Broomfield and Dodd (2004).

Associations have been shown between lower language levels and birth order or family size (Choudhury & Benasich, 2003; Harrison & McLeod, 2010; Reilly et al., 2007; Stanton-Chapman, Chapman, Bainbridge, & Scott, 2002; J. B. Tomblin, Hardy, & Hein, 1991; Zubrick, Taylor, Rice, & Slegers, 2007), overcrowding in homes (Law et al., 2009), absence of preschool education, and parental language and literacy levels (Eadie et al., 2015; Law et al., 2009). With regard to languages spoken in the child's environment, some have found that children are more likely to be identified as speech or language impaired when the language spoken at home is different from that spoken out of the home (Reilly et al., 2007, 2010), whereas others have found the reverse (Broomfield & Dodd, 2004; Harrison & McLeod, 2010; Stanton-Chapman et al., 2002).

Family and environmental factors extend to the pre- and perinatal factors studied by Delgado et al. (2005), Fox et al. (2002), and Wolke and Meyer (1999), as these factors relate to the medical status into which the child is born. Their studies produced mixed findings, with some pre- and perinatal factors showing a positive association with SSD.

### Developmental Progression in Speech and Language

Although one study did not observe a relationship between early language skills and later speech (Broomfield & Dodd, 2004), delay in early language development generally has been positively associated with SSD (Eadie et al., 2015; Highman, Hennessey, Sherwood, & Laitao, 2008) and with speech and language impairment (Bishop & Edmundson, 1987; Dale, Price, Bishop, & Plomin, 2003; Glogowska, Roulstone, Peters, & Enderby, 2006; Rescorla, 2002; Rice, Taylor, & Zubrick, 2008; Roulstone, Miller, Wren, & Peters, 2009). Moreover, the relationship between language development and SSD appears to remain relatively constant over time, with studies of school-age factors showing a similar pattern (Bishop & Adams, 1990; Broomfield & Dodd, 2004; Lewis et al., 2006; Raitano, Pennington, Tunick, Boada, & Shriberg, 2004; Shriberg et al., 1999).

### Developmental Progression in Literacy and Learning

Studies that have focused on school-age factors have often considered the relationship between SSD and literacy skills. Indeed, given the association observed, there has been much debate about whether literacy skill should be regarded as an outcome of SSD or whether the two are part of the same underlying condition (Bird, Bishop, & Freeman, 1995; Felsenfeld, Broen, & McGue, 1994; Gillon & Moriarty, 2007; Hesketh, 2004; Larrivee & Catts, 1999; Leitao, Fletcher, & Hogben, 2000; Nathan, Stackhouse, Goulandris, & Snowling, 2004; Peterson, Pennington, Shriberg, & Boada, 2009; Raitano et al., 2004; Rvachew, 2007; Sutherland & Gillon, 2007).

### Other Developmental Factors

Beyond speech-language and literacy or learning development, other areas of development that have shown associations with SSD and/or language skills include use of pacifiers (Fox et al., 2002); delay in motor skills, including feeding and dribbling (Eadie et al., 2015; Highman et al., 2008; Hill, 2001; Hill & Bishop, 1998; Robinson, 1991; Visscher, Houwen, Scherder, Moolenaar, & Hartman, 2007; Visscher et al., 2010; Webster, Majnemer, Platt, & Shevell, 2005); general delays and medical conditions (Broomfield & Dodd, 2004; Delgado et al., 2005); and low birth weight (Stanton-Chapman et al., 2002; Yliherva, Olsén, Mäki-Torkko, Koiranen, & Järvelin, 2001). With regard to hearing and ear, nose, and throat status, mixed findings have emerged, with some studies showing a relationship with SSD and others suggesting that none exists (Browning, Rovers, Williamson, Lous, & Burton, 2010; Campbell et al., 2003; Fox et al., 2002; Pagel Paden, 1994). Indeed, the findings of Paradise et al. (2005, 2007) from a large-scale longitudinal study suggest that otitis media with effusion and associated hearing loss are not associated with SSD in otherwise healthy individuals.

In conclusion, the information from these studies provides a challenging picture for the clinician to interpret. None of the studies provide a comprehensive analysis of a range of potential variables and their relative importance in relation to predicting persistent SSD. However, the examination of the literature has generated putative factors that may be associated with persistent SSD. These have been used to establish a comprehensive model of risk encompassing demographic, environmental, and developmental components of the child's history and characteristics. Data from large-scale population-based studies offer the opportunity to study associations between a variety of potential predictor variables and later speech outcomes while controlling for other confounding developmental and social factors (Roulstone, Law, Rush, Clegg, & Peters, 2011). The study reported in this article uses data from the Avon Longitudinal Study of Parents and Children (ALSPAC), a prospective population study taking place in the southwest of England. This large study has collected detailed data on children's speech and language at several time points through direct assessment along with a wide range of developmental, environmental, and social data on the children and their families. This unique data set enables relatively comprehensive consideration of confounding effects in developing the risk model through taking account of the relationships between such a wide variety of variables.

Numerous articles on a range of health and developmental factors have reported on the ALSPAC data to date, including five on findings relating to children's speech and language development (Roulstone et al., 2009, 2011; Wren, 2015; Wren, McLeod, White, Miller, & Roulstone, 2013; Wren, Roulstone, & Miller, 2012). With regard to speech development and disorder, results from an analysis of the longitudinal data on a subset of the children (*n* = 741) at ages 2, 5, and 8 years show a relationship between the child's speech error rates at ages 2 and 5 years and expressive language. SSD at age 8 years was predicted by presence of speech errors at age 5 years but not at age 2 years (Roulstone et al., 2009). Further analysis has been reported on the characteristics of the sample in terms of speech production (Wren et al., 2013) and features that distinguish the groups identified through the process of case identification described in this article (Wren et al., 2012). The purpose of the study reported in this article was to use the data available from this large-scale population cohort to investigate persistent SSD and factors associated with it that could be used to estimate prevalence and to identify predictor variables that could assist clinicians in identifying young children at risk of persistent SSD and aid our understanding of the nature of persistent SSD.

## Aim

The aim of this study was to use direct assessment to identify children with persistent SSD at age 8 years. Following identification, the objectives were (a) to determine the prevalence of persistent SSD in children aged 8 years and (b) to identify early childhood and later school-age social, cognitive, and linguistic predictors that are associated with a classification of persistent SSD at age 8 years.

## Method

### ALSPAC

This study used prospective cohort data from ALSPAC, a transgenerational observational population study of health and development across the life span. Multiple measures of genetic, epigenetic, biological, psychological, social, and other environmental factors have been collected in relation to outcomes. A description of the cohort profile is available (Boyd et al., 2012). In 1991 and 1992, 14,541 mothers enrolled in ALSPAC as they registered their pregnancy in the geographical area then known as Avon in the southwest of the United Kingdom. Out of the initial 14,541 pregnancies, 14,062 live babies were born and 13,988 children were alive at 1 year.

The main data collection technique for the study has been postal surveys: The mothers completed four questionnaires before their babies were born and approximately annually thereafter, with 16 surveys completed by the time the child was aged 13 years. In addition, since the children were aged 7 years, the entire cohort was invited to attend for direct assessment of varying aspects of development at regular intervals (known as the *focus clinics*). The second of these focus clinics was the “Focus at 8” clinic, in which speech and language were assessed.

The study website includes details of all the data that are available through a fully searchable data dictionary (http://www.bris.ac.uk/alspac/researchers/data-access/data-dictionary/). Ethical approval for the study was obtained from the ALSPAC Law and Ethics Committee and the local research ethics committees.

### Participants

Participants in this study were children who completed the speech and language session at the Focus at 8 clinic. All 13,314 children from the cohort who were still alive and consenting and who had known addresses were invited to attend this clinic, and appointments were arranged for when the children were aged 8 years 6 months. A total of 7,391 children (56%) attended, though records for one child were incomplete and the child's data were therefore excluded from any further analysis. The sample of children who attended was biased in that it contained a significantly greater proportion of higher educated and older mothers who were more likely to be living in owner-occupied housing. A slightly smaller proportion of boys and non-White children attended compared with nonattendees. Children who attended also had a slightly higher mean birth weight, but there was no difference in mean gestation. It is worth noting, however, that with the size of the sample there were still many people in each category of the categorical variables and across the spectrum of the continuous variables.

The sample was heterogeneous in that it included all children who completed the speech and language session during the Focus at 8 clinic. Children were not excluded if they had comorbid conditions such as cerebral palsy, hearing impairment, cleft palate, learning difficulties, or any other condition that could have affected or caused their speech development. Data on the numbers of children in the sample who presented with comorbid conditions are variable and incomplete and therefore unreliable. However, as a population sample, it could be assumed that prevalence of comorbid conditions within the sample would likely match that for the U.K. population as a whole. Likewise, attempts were not made to classify the sample into subgroups on the basis of surface-level speech errors or into children with speech impairment only versus children with both speech and language impairment. Rather, this article reports on the group as a whole. It is anticipated that further research will be carried out in the future to consider the impact of speech impairment only versus speech and language impairment.

### Speech Sampling

At the Focus at 8 clinic, connected speech samples were collected during an expressive language task on the basis of the Wechsler Objective Language Dimensions (Rust, 1996). In this activity, three tasks were performed: picture description, giving directions using a map, and explaining the steps involved in changing the batteries in a flashlight. All responses in this task were recorded digitally.

### Identification of Cases of Persistent SSD

The process of case identification for persistent SSD within the cohort consisted of three phases:

1. Listener judgment. Assessors noted children whose speech sounded atypical for their age and whose errors were inconsistent with the local accent during the speech and language assessment. Children were assessed by qualified speech-language pathologists (85.9%) or psychologists trained by a speech-language pathologist in the delivery of the assessments (14.1%). Those children whose errors, as observed by assessors, were limited solely to common clinical distortions as defined by Shriberg (1993) were identified. In the United Kingdom, children with these types of errors typically are not seen for intervention at this age, and for this reason they were excluded from the definition of persistent SSD. The remaining children—those showing a range of substitution, omission, addition, and atypical distortion errors with or without the common clinical distortions—were considered potential cases.
2. Transcription. All sounds within the connected speech samples of the potential case group were transcribed and analyzed using Computerized Profiling (Long, Fey, & Channell, 2006). Broad transcription was used for sounds that were perceptually correct and for whole-sound substitutions, omissions, and additions, whereas atypical distortion errors were narrowly transcribed. A further 50 speech samples were transcribed from children who were randomly selected from the rest of the cohort (25 boys, 25 girls) to act as controls for the purpose of calculating prevalence. Transcribers were blind to the status of the sample being transcribed and were qualified speech-language pathologists.
3. Comparison with controls. Means and standard deviations for the percentage consonants correct (PCC) late eight (/s, z, ʃ, ʒ, θ, ð, ɹ, l/; PCC late 8) and PCC adjusted (PCC-A) measures (Shriberg, Austin, Lewis, McSweeny, & Wilson, 1997a) were calculated for the 50 control children. PCC is a measure of speech accuracy in which the number of correctly produced consonants is counted and calculated as a percentage of the total target number of consonants in the sample. Given the age of the children, the PCC late 8 was considered to be more sensitive than total PCC. The PCC-A was selected because this measure accepts common clinical distortions as correct but not atypical distortions, thus matching the criteria with which the children were selected in phase 1.

Means and standard deviations were calculated separately for girls and boys and used to identify cases. Using the control group as a reference, potential cases were classified as persistent SSD if they scored less than 1.2 *SD*s below the mean on both the PCC late 8 and the PCC-A. This cutoff was selected for consistency with Records and Tomblin's (1994) observations that clinicians' decisions regarding diagnosis was associated with a cutoff composite score of approximately −1.2 *SD*s.

Thus, the criteria for categorization of persistent SSD in this study was a score of less than 1.2 *SD*s below the mean of the control group on both the PCC late 8 and the PCC-A on connected speech samples taken during picture description tasks. The data for these case children were used in comparison with the rest of the cohort (*n* = 6,399) in subsequent analyses to identify early childhood and school-age predictors. The two groups of children identified exclusively with common clinical distortions and the group of potential cases who did not reach criteria for case status (i.e., ≥1.2 *SD*s below the means for either the PCC late 8 or the PCC-A) were excluded from this analysis. A separate analysis revealed that these latter two groups showed distinct features in terms of demographic factors, IQ, nonword repetition, and diadochokinetic (DDK) tasks compared with the case children and those in the rest of the cohort (Wren et al., 2012). Inclusion of their data could therefore have contaminated findings in the analyses carried out in this study.

A randomly selected sample of 48 children was transcribed by a second member of the original transcription team to check reliability. Point-to-point interjudge agreement was 92.3%. As reliability was completed post hoc, it was not possible to resolve discrepancies, and the first transcription was used in the analysis.

### Identification of Candidate Predictor Variables for Persistent SSD

The ALSPAC data source was investigated to identify predictors potentially associated with persistent SSD on the basis of the literature summarized in the Introduction. Potential predictors were grouped into early childhood and school-age predictor variables and analyzed separately. Early childhood predictors were those collected between the prenatal and immediate postbirth period up to the age of the school entry assessments.^1^ The one exception to this was the data relating to the range of languages spoken in the home, which was included in a questionnaire to the mothers when the children were aged 6 years 9 months. However, the data relating to this question were included in the early childhood group because the impact would occur from birth. School-age predictor variables were those that were collected between the ages of 5 years 9 months and 8 years 7 months. The exception to this was the demographic variables, which were included in the analysis of both early childhood and school-age predictors as potential confounding variables.

Tables 4 and 5 list the variables included in the categories of early childhood and school-age predictors, respectively, along with the timing and method of data collection. They were grouped conceptually for later analysis within each of the two categories. Further details on all the variables included in the analysis are available in the online supplemental materials (see Supplemental Tables 1 and 2).

**Table 4.** Summary of demographic and early childhood candidate predictor variables included in the analysis.

Grouped variable	Variable	Method of data collection	Timing of data collection
Demographic	Gender (categorical: boy/girl)	Birth records from midwife	Birth
	Ethnicity (categorical: White/non-White)	Questionnaire to mother	32 weeks gestation
	Level of maternal education^a^ (categorical: < O level/O level/> O level)	Questionnaire to mother	32 weeks gestation
	Maternal occupation (categorical: nonmanual/manual)	Questionnaire to mother (supplemented with information on father if information on maternal occupation was not available)	32 weeks gestation
	Home ownership (categorical: mortgaged or owned/rented or other)	Questionnaire to mother	8 weeks gestation
	Maternal age at birth of child (continuous)	Midwife records	Recruitment to study
Environment	Parity (i.e., how many previous pregnancies resulted in a live birth or stillbirth; categorical: first child, second child, third or more child)	Questionnaire to mother	32 weeks gestation
	Languages other than English used at home (categorical: yes/no)	Questionnaire to mother	Child aged 81 months
	Preschool provision: Child attends day nursery/crèche (categorical: yes/no)	Questionnaire to mother	Measure repeated when child was aged 8 weeks, 8 months, 15 months, 24 months, 38 months, and 54 months
	Preschool provision: Child attends nursery, playgroup, or childminder (categorical: yes/no)	Questionnaire to mother	Child aged 33 months and 47 months
	Reading to the child (categorical: almost daily, three to five times per week, less than three times per week)	Questionnaires to mother	Child aged 18 months (mother and partner) and 24 months (mother only)
	Reading to the child (categorical: almost daily, one to five times per week, less than once per week)	Questionnaires to mother	Child aged 42 months (mother, partner, and other person)
	Overcrowding (categorical: < 0.50 person per room, 0.50 to 0.75 person per room, 0.75 to 1.00 person per room, > 1.00 person per room)	Questionnaires to mother	8 weeks gestation and when child was aged 21 months and 33 months
	Family history (categorical: yes/no)	Questionnaires to mother and partner	12 weeks gestation
	Premature birth (categorical: yes/no)	Medical records	Postbirth
	Method of delivery (categorical: spontaneous, assisted, elective caesarean, emergency caesarean)	Medical records	Postbirth
	Pregnancy complications: hypertension, unexplained abdominal pain, vaginal bleeding, vomiting, any complication (categorical: yes/no)	Medical records	Postbirth
	Breastfeeding (categorical: never, < 3 months, > 3 months)	Questionnaire to mother	Child aged 6 months
	Smoking: at any time, prepregnancy, during first trimester, during last 2 weeks (categorical: yes/no)	Questionnaire to mother	18 weeks gestation
	First child as a teenager (categorical: yes/no)	Questionnaire to mother	18 weeks gestation
	Feelings (continuous)	Questionnaires to mother	8 weeks and 32 weeks gestation and when the child was aged 8 weeks, 8 months, 21 months, and 33 months
Early speech and language performance	MacArthur Communicative Development Inventories (adapted)^b^ understanding and saying vocabulary (continuous)	Questionnaire to mother	Child aged 38 months
	Intelligibility to mother, family, and others (categorical: mostly/sometimes or rarely)	Questionnaire to mother	Child aged 38 months
	Use of gesture (categorical: no, never did/yes but not now/yes and still does)	Questionnaire to mother	Child aged 38 months
	Word combination (categorical: often/sometimes/not yet)	Questionnaire to mother	Child aged 24 months and 38 months
	Word morphology (continuous)	Questionnaire to mother	Child aged 38 months
	Irregular grammar (continuous)	Questionnaire to mother	Child aged 24 months
	Stuttering (categorical: never/sometimes/often)	Questionnaire to mother	Child aged 38 months
	Denver Communication Score (continuous)	Questionnaires to mother	Child aged 6 months and 18 months
Early literacy and learning skills	School entry assessments: reading (categorical: achieved expected level/exceeded expected level)	School entry assessments	Entry to school at age 4 to 5 years
	School entry assessments: writing (categorical: achieved expected level/exceeded expected level)	School entry assessments	Entry to school at age 4 to 5 years
Other early developmental variables	Low birth weight (categorical: < 2500 g/≥ 2500 g)	Medical records	Postbirth
	Avon Longitudinal Study of Parents and Children developmental scale: social, fine motor, and gross motor scales (continuous)	Questionnaires to mother	Child aged 6 months, 18 months, 30 months, and 42 months
	School entry assessments: large and fine motor (categorical: achieved expected level/exceeded expected level)	School entry assessments	Entry to school at age 4 to 5 years
	Laterality (categorical: right/mixed/left)	Questionnaire to mother	Child aged 42 months
	Feeding difficulties (categorical: yes/no)	Questionnaire to mother	Child aged 4 weeks

*Note.* Comments in parentheses indicate whether the variable is categorical (with specified categories) or continuous.

a “O level” was the qualification obtained at age 16 years when the parents of the cohort were at school.

b A reduced version of the Communicative Development Inventories was used due to time taken to complete the questionnaire (which covered a range of topics) and space for printing (Fenson et al., 1993).

**Table 5.** Summary of demographic and school-age candidate predictor variables included in the analysis.

Grouped variable	Specific variable	Method of data collection	Timing of data collection^a^
Demographic	Gender (categorical: boy/girl)	Birth records from midwife	Birth
	Ethnicity (categorical: White/non-White)	Questionnaire to mother	32 weeks gestation
	Level of maternal education^b^ (categorical: < O level/O level/> O level)	Questionnaire to mother	32 weeks gestation
	Maternal occupation (categorical: nonmanual/manual)	Questionnaire to mother (supplemented with information on father if information on maternal occupation was not available)	32 weeks gestation
	Home ownership (categorical: mortgaged or owned/rented or other)	Questionnaire to mother	8 weeks gestation
	Maternal age at birth of child (continuous)	Midwife records	Recruitment to study
Later speech and language performance	Language comprehension (continuous)	Listening Comprehension subtest of Wechsler Objective Language Dimensions Part II	Focus at 8
	Diadochokinetic tasks (a measure of oral motor skill; categorical: correct/incorrect)	Repetition of a variety of syllables (pe, te, ke, peteke, bedege) for 10 s each (Northstone et al., 2006)	Focus at 8
	Phoneme deletion (continuous)	Auditory Analysis Test (Rosner & Simon, 1971)	Focus at 7
	Difficulty pronouncing sounds (categorical: yes/no)	Questionnaire to mother	Child aged 81 months
	Nonword repetition (continuous)	CNRep (adapted; 12 items: four each of three, four, and five syllables; Gathercole & Baddeley, 1994)	Focus at 8
Literacy and learning performance	Reading test (continuous)	WORD Reading subtest (Rust et al., 1993)	Focus at 7
	Spelling test (continuous)	SpellingTest (15 words; Northstone et al., 2005)	Focus at 7
	School assessment: reading (categorical: achieved expected level/underachieved/exceeded expected level)	Key Stage 1 Standard Attainment Tests	End of year 2 in U.K. primary school (child aged 6–7 years)
	School assessment: writing (categorical: achieved expected level/underachieved/exceeded expected level)	Key Stage 1 Standard Attainment Tests	End of year 2 in U.K. primary school (child aged 6–7 years)
	Identified learning problem (categorical: yes/no)	Questionnaire to mother	Child aged 77 months
Other developmental variables	Verbal IQ (continuous)	WISC-III UK (reduced form using alternate test items)	Focus at 8
	Performance IQ (continuous)	WISC-III UK (reduced form using alternate test items)	Focus at 8
	Combined IQ score (continuous)	WISC-III UK (reduced form using alternate test items)	Focus at 8
	Auditory memory (continuous)	Digit Span subtest of WISC-III UK (reduced form using alternate test items)	Focus at 8
	Spatial ability (continuous)	Block Design subtest of WISC-III UK (reduced form using alternate test items)	Focus at 8
	Attention (continuous)	Sky Search task from TEACh (Manly et al., 1998)	Focus at 8
	Friendships (continuous)	Friendships questionnaire (Goodyer et al., 1989, 1990)	Focus at 8
	Suspected coordination problem (categorical: yes/no)	Questionnaire to mother	Child aged 103 months
	Tympanostomy tubes fitted at any time (categorical: yes/no)	Questionnaires to mother and hearing assessment	Child aged 69 months and 81 months (questionnaires); Focus at 7 (hearing assessment)
	Hearing impairment (categorical: yes/no)	Pure-tone audiometry	Focus at 7

*Note.* Comments in parentheses indicate whether the variable is categorical (with specified categories) or continuous. CNRep = Children's Test of Non-Word Repetition; WORD = Wechsler Objective Reading Dimensions; WISC-III UK = Wechsler Intelligence Scale for Children–Third UK Edition (Wechsler, Golombok, & Rust, 1992); TEACh = Test of Everyday Attention for Children.

a “Focus” is the name of the assessment clinics that children from the Avon Longitudinal Study of Parents and Children sample were invited to attend. “Focus at 8” is the name of the clinic that children attended at age 8 years; “Focus at 7” is the name of the clinic that children attended at age 7 years.

b “O level” was the qualification obtained at age 16 years when the parents of the cohort were at school.

### Statistical Analysis

Following identification of the case group, the prevalence of persistent SSD in the sample of children who attended the Focus at 8 clinic and for whom data were available was calculated. Following appropriate descriptive statistics (means, standard deviations, and proportions), univariable and multivariable logistic regression analyses (Peters, 2008) were used to obtain odds ratios (ORs), 95% confidence intervals (CIs), and likelihood ratio *p* values for the associations between persistent SSD and various early childhood and school-age predictor variables. Both continuous and categorical explanatory variables were used in the analysis. The first stage of analysis tested all variables for their association with the outcome variable—that is, the child's case status at 8 years. Variables with a *p* value of < .10 in univariable analyses were retained for use in the multivariable analyses. A deliberately tolerant level was used in order to not miss any potentially influential variables at this point, whereas *p* < .05 was used in all the multivariable analyses. In addition, maternal age was retained in all regression models owing to evidence of its possible contribution in a related study using the same data set (Roulstone et al., 2009).

A staged multivariable regression approach (Patel, Peters, Murphy, & the ALSPAC Study Team, 2005) was then used, first within the groups of conceptual variables (as listed in Tables 4 and 5) and then across groups. This resulted in a final model of demographic and early childhood and school-age predictors independently associated with case status.

At each step in this process, only one variable was dropped from or added to the model at any one point in order to ensure that all independent influences on the outcome were retained. In the final stage of analysis, variables from the within-group multivariable analyses that were associated with case status (*p* < .05) were combined into two final models of predictors associated with case status (early childhood and school-age predictors). This between-groups model was adjusted for the child's gender and social class and for maternal age.

Given the nature of the study and the number of variables collected, there were missing data at various points in the analysis. In each analysis, we worked with the maximum data available for the variables under investigation. All analyses were conducted in Stata (Version 13 Stata Corp, Texas, USA).

## Results

### Prevalence of Persistent SSD

**Figure 1.**
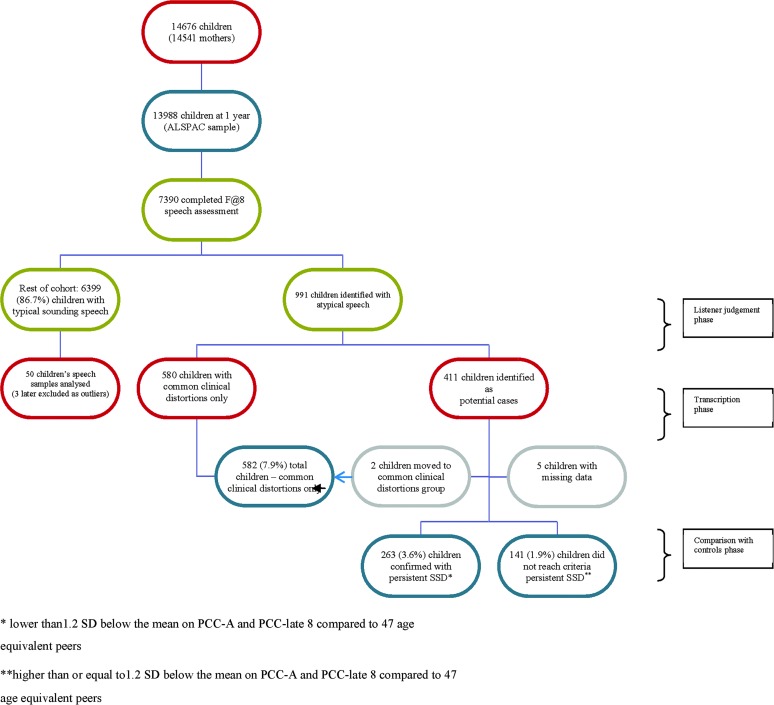
Summary of case identification. ALSPAC = Avon Longitudinal Study of Parents and Children; SSD = speech sound disorder.

Figure 1 summarizes the process of case identification. Of the 7,390 children who had data from the Focus at 8 speech and language assessment, 991 children had speech that sounded immature or unusual for their age and errors that were inconsistent with the local accent during the listener judgement phase. From the remaining 6,399 children whose speech sounded typical for their age and accent, 50 were selected at random as a control group. The data for three of the control children were markedly outside the range of the data for the remaining 47 controls—specifically, PCC-A scores of 71.9, 74.0, and 77.4 compared with a range of 94.7 to 100.0 for the remaining controls. Because inclusion of these children's data would have markedly altered the standard deviation cutoffs for the identification of the case group, their data were not used to calculate means and standard deviations for the control sample. However, these data, along with data for the rest of the cohort, were used in the regression analyses for identification of predictor variables. Table 6 provides the means and standard deviations for both the PCC late 8 and the PCC-A for the 47 controls as well as the calculation of the −1.2 *SD*s cutoff for each measure.

**Table 6.** Means, standard deviations, and cutoff scores for measures of connected speech in the control group.

Variable	Controls (*n*)	*M (SD)*	Cutoff score
PCC late 8^a^			
Males	24	95.8% (4.3)	90.7%
Females	23	97.6% (3.6)	93.2%
Total	47	96.7% (4.0)	91.9%
PCC adjusted^b^			
Males	24	97.8% (1.6)	95.8%
Females	23	98.5% (1.7)	96.5%
Total	47	98.1% (1.7)	96.1%

*Note.* Cutoff score = −1.2 *SD*. PCC = percentage consonants correct.

a The eight consonants that are acquired last in a typical developmental sequence (/s, z, ʃ, ʒ, θ, ð, ɹ, and l/).

b The percentage of consonants correctly produced, excluding common clinical distortions (Shriberg, 1993).

Of the 991 children who were identified through listener judgment as showing speech that was atypical for their age and accent, 580 made common clinical distortions exclusively (Shriberg, 1993). The remaining 411 showed a range of whole-sound substitutions, omissions, atypical distortions, and additions with or without common clinical distortions as described previously.

Within the sample of 411 potential cases, five cases were removed from all analyses due to missing speech samples. The rest of their data were removed from the study for all further analyses. For the remaining 406 children, PCC late 8 and PCC-A scores from the transcribed connected speech samples were compared with those obtained from the 47 controls and used to confirm cases of persistent SSD.

Two children within the potential case group had a PCC-A score of 100% and a PCC score of less than 100%. This would suggest that all their errors were distortions of sibilants and rhotics (PCC-A scores all speech errors, including common clinical distortions, as correct, whereas PCC scores them as incorrect). They were therefore added to the group of children previously identified as showing only common clinical distortions, taking the total in this group to 582. Therefore, 582 out of 7,385 (total cohort of 7,390 minus five with missing data), or 7.9% of the cohort, 95% CI [7.3, 8.5], presented with common clinical distortions (see Figure 1).

Of the 404 remaining children identified as potential cases through listener judgment, 263 (169 boys, 94 girls) were confirmed as cases on the basis of cutoff values derived from PCC late 8 and PCC-A scores obtained from the 47 control children. From a total sample size of 7,385, 263 cases yields an estimated prevalence of 3.6% overall, 95% CI [3.1, 4.0]. In terms of gender, this equates to a prevalence of 4.6% for boys (on the basis of a total sample of 3,687 boys) and 2.5% for girls (total sample of 3,698 girls), giving a ratio of 1.8:1.

Table 7 shows the descriptive statistics for the PCC late 8 and PCC-A scores across the three groups (controls, confirmed cases of persistent SSD, and potential cases who did not reach the criteria for case status). This confirms that children with persistent SSD had a lower mean and larger standard deviation for each measure, although there is some overlap in the ranges for both PCC late 8 and PCC-A for all three groups.

**Table 7.** Descriptive statistics for measures of connected speech for control children, confirmed cases of persistent speech sound disorder, and potential cases who did not reach criteria for case status.

Group	*n*	PCC late 8^a^		PCC-A^b^	
		*M* (*SD*)	Range	*M* (*SD*)	Range
Controls	47	96.7 (4.0)	85.2–100.0	98.1 (1.7)	94.7–100.0
Confirmed cases of persistent speech sound disorder (<1.2 *SD* below the mean on both PCC late 8 and PCC-A)	263	70.5 (15.5)	24.1–93.2	87.8 (7.0)	42.1–96.4
Potential cases who did not reach criteria for case status (≥1.2 *SD* below the mean on either PCC late 8 or PCC-A)	141	95.4 (4.2)	71.7–100.0	97.0 (2.3)	87.3–100.0

*Note.* PCC = percentage consonants correct.

a The eight consonants that are acquired last in a typical developmental sequence (/s, z, ʃ, ʒ, θ, ð, ɹ, and l/).

b The percentage of consonants correctly produced, excluding common clinical distortions (Shriberg, 1993).

### Predictors Associated With Persistent SSD

The regression analyses were conducted using maximum numbers of 263 children with confirmed persistent SSD and the 6,399 children composing the rest of the cohort (including all 50 of those who had been selected randomly as controls and whose samples had been transcribed). For the final across-groups analysis, a total sample size of 5,066 children (out of a possible 6,662) was available for the early childhood predictor variables, and a sample size of 4,303 children was available for the school-age predictor variables.

In univariable analysis, compared with the rest of the cohort, case children were more likely to be boys, to have mothers who were less well educated and in manual professions, and to live in rented homes. These sociodemographic factors were then considered separately for the early childhood and school-age predictor variables alongside the other grouped variables in a staged process of within-group and between-groups multivariable regression models that were reduced using a manual forward and backward stepwise process. The results of the univariable analysis are available in the online supplemental materials (see Supplemental Tables 3 and 4).

### Early Childhood Predictors

Factors with *p* values greater than .10 following univariable analysis were ethnicity, maternal age, attendance at preschool provision up to age 33 months and at 54 months, reading to the child at age 42 months, preterm delivery, pregnancy complications (except vaginal bleeding), method of labor, breastfeeding, smoking in early pregnancy or prepregnancy, teenage motherhood, maternal depression and anxiety, stuttering at 38 months, communication, social and gross motor scores at 6 months, low birth weight, laterality at 42 months, and various feeding factors at 4 weeks. These factors were excluded from further analysis.

Table 8 lists the variables for which the *p* value following univariable regression was less than .10. These variables were taken forward to the within-group multivariable analysis. From these analyses, 13 variables (gender, maternal occupation,^2^ home ownership, mother reading to child at 18 months, overcrowding at 8 weeks, family history of referral to speech and language therapy, intelligibility to others, combining words at 24 months, use of irregular grammar at 24 months, range of word morphology at 38 months, fine motor skills at 42 months, gross motor skills at 42 months, and weak sucking and dribbling at 4 weeks) showed some evidence of association (*p* < .05) at this stage (see Table 9). All of these except maternal social class (*p* = .014) were retained in the between-groups multivariable analysis stage to end with the variables listed as the “best” model.

**Table 8.** Descriptive statistics and univariable regression model results for demographic and early childhood risk factor variables associated with persistent speech sound disorder, where *p* < .10 (with maternal age and gender included regardless of their *p* values).

Grouped variable^a^	Category^b^	Total sample (*N*)	Case children summary data^c^	Rest of cohort summary data^c^	Univariable model		*p* value
					Odds ratio	95% confidence intervals	
Demographics							
Gender^d^	Female	6,662	94 (2.8)	3,303 (97.2)	1.00		<.001
	Male		169 (5.2)	3,096 (94.8)	1.92	[1.48, 2.48]	
Level of maternal education^d^^,^ ^e^	O level	6,166	79 (3.6)	2,093 (96.4)	1.00		.025
	< O level		70 (5.2)	1,265 (94.8)	1.47	[1.05, 2.04]	
	> O level		93 (3.5)	2,566 (96.5)	0.96	[0.71, 1.30]	
Maternal occupation^d^^,^ ^f^	Nonmanual	5,909	156 (3.3)	4,616 (96.7)	1.00		<.001
	Manual		64 (5.6)	1,073 (94.4)	1.76	[1.31, 2.38]	
Home ownership^d^	Mortgaged/owned	6,199	174 (3.4)	5,007 (96.6)	1.00		<.001
	Rented/other		70 (6.9)	948 (93.1)	2.12	[1.60, 2.83]	
Maternal age^g^		6,382	29.2 (4.8)	29.1 (4.6)	1.01	[0.98, 1.03]	.72
Environment							
Parity^d^	First child	6,161	84 (2.9)	2,805 (97.1)	1.00		
	Second child		100 (4.6)	2,068 (95.4)	1.61	[1.20, 2.17]	<.001
	Third or more child		55 (5.0)	1,049 (95)	1.75	[1.24, 2.48]	
Languages other than English used in the home^d^	No	5,399	194 (3.8)	4,959 (96.2)	1.00		.024
	Yes		17 (6.9)	229 (93.1)	1.90	[1.14, 3.17]	
Child attends day nursery regularly at age 38 months^d^	No	5,770	150 (4.2)	3,441 (95.8)	1.00		
	Yes		72 (3.3)	2,107 (96.7)	0.78	[0.59, 1.04]	.091
Child attends playgroup, nursery, or childminder at age 47 months^d^	No	5,583	13 (8.1)	148 (91.9)	1.00		.013
	Yes		203 (3.7)	5,219 (96.3)	0.44	[0.25, 0.79]	
Mother reads to child at age 18 months^d^	Almost daily	5,973	148 (3.4)	4,219 (96.6)	1.00		.003
	Three to five times per week		52 (4.7)	1,050 (95.3)	1.41	[1.02, 1.95]	
	Less than three times per week		32 (6.3)	472 (93.7)	1.93	[1.30, 2.87]	
Partner reads to child at age 18 months^d^	Almost daily	5,750	64 (3.2)	1,929 (96.8)	1.00		.035
	Three to five times per week		51 (3.5)	1,412 (96.5)	1.09	[0.75, 1.58]	
	Less than three times per week		107 (4.7)	2,187 (95.3)	1.47	[1.08, 2.02]	
Either parent reads to child at age 18 months^d^	Almost daily	5,984	126 (3.4)	3,593 (96.6)	1.00		.012
	Three to five times per week		66 (4.4)	1,421 (95.6)	1.48	[1.06, 2.09]	
	Less than three times per week		34 (5.8)	555 (94.2)	1.77	[1.11, 2.83]	
Mother reads to child at age 24 months^d^	Almost daily	5,795	168 (3.5)	4,612 (96.5)	1.00		.013
	Three to five times per week		44 (5.1)	814 (94.9)	1.32	[0.98, 1.80]	
	Less than three times per week		21 (6.1)	325 (93.9)	1.75	[1.18, 2.58]	
Overcrowding index at 8 weeks of gestation^d^^,^ ^h^	≤ 0.50	6,132	90 (3.0)	2,913 (97.0)	0.10		<.001
	0.50–0.75		80 (4.2)	1,834 (95.8)	1.41	[1.04, 1.92]	
	0.75–1.00		45 (4.6)	929 (95.4)	1.57	[1.09, 2.26]	
	> 1.00		22 (9.1)	219 (90.9)	3.25	[2.00, 5.29]	
Overcrowding index at age 21 months^d^^,^ ^h^	≤ 0.50	5,519	37 (3.0)	1,180 (97.0)	1.00		.006
	0.50–0.75		71 (3.2)	2,152 (96.8)	1.05	[0.70, 1.58]	
	0.75–1.00		46 (5.0)	875 (95.0)	1.68	[1.08, 2.61]	
	> 1.00		59 (5.1)	1,102 (94.9)	1.71	[1.12, 2.60]	
Overcrowding index at age 33 months^d^^,^ ^h^	≤ 0.50	5,501	40 (3.7)	1,052 (96.3)	1.00		.083
	0.50–0.75		70 (3.2)	2,108 (96.8)	0.87	[0.59, 1.30]	
	0.75–1.00		91 (4.8)	1,817 (95.2)	1.32	[0.90, 1.93]	
	> 1.00		12 (3.7)	311 (96.3)	1.01	[0.53, 1.96]	
Family history of speech and language therapy attendance^d^	No	6,135	218 (3.7)	5,642 (96.3)	1.00		.007
	Yes		20 (7.3)	255 (92.7)	2.03	[1.26, 3.26]	
Pregnancy complications: vaginal bleeding^d^	No	6,662	231 (3.8)	5,822 (96.2)	1.00		.096
	Yes		32 (5.3)	577 (94.8)	1.40	[0.96, 2.04]	
Smoked during last 2 weeks of pregnancy^d^	No	6,255	196 (3.6)	5,189 (96.4)	1.00		.017
	Yes		47 (5.4)	823 (94.6)	1.51	[1.09, 2.10]	
Early speech and language performance							
MacArthur understanding vocabulary score at 38 months^g^		5,770	10.3 (2.6)	11.1 (2.3)	0.91	[0.87, 0.95]	<.001
MacArthur saying vocabulary score at 38 months^g^		5,770	9.9 (3.2)	11.4 (1.6)	0.77	[0.73, 0.80]	<.001
Intelligibility to mother at 38 months^d^	Mostly	5,714	197 (3.5)	5,378 (96.5)	0.10		<.001
	Sometimes/rarely		23 (16.6)	116 (83.5)	5.41	[3.39, 8.66]	
Intelligibility to family at 38 months^d^	Mostly	5,712	150 (2.9)	5,051 (97.1)	0.10		
	Sometimes/rarely		68 (13.3)	443 (86.7)	5.17	[3.82, 6.99]	<.001
Intelligibility to others at 38 months^d^	Mostly	5,703	111 (2.4)	4,526 (97.6)	0.10		
	Sometimes/rarely		107 (10.0)	959 (90.0)	4.55	[3.46, 5.99]	<.001
Uses gestures at or before 38 months^d^	No, never did	5,696	37 (2.2)	1,632 (97.8)	1.00		<.001
	Yes but not now		127 (3.5)	3,465 (96.5)	1.62	[1.12, 2.34]	
	Yes and still does		57 (13.1)	378 (86.9)	6.65	[4.33, 10.2]	
Word combination at 24 months^d^	Often	5,628	66 (2.0)	3,171 (98.0)	1.00		<.001
	Sometimes		74 (4.8)	1,462 (95.2)	2.43	[1.74, 3.41]	
	Not yet		72 (8.4)	783 (91.6)	4.42	[3.14, 6.23]	
Word combination at 38 months^d^	Often	5,641	174 (3.3)	5,121 (96.7)	1.00		<.001
	Sometimes		25 (8.7)	262 (91.3)	2.81	[1.81, 4.35]	
	Not yet		18 (30.5)	41 (69.5)	12.9	[7.28, 22.9]	
Word morphology at 38 months^g^		5,711	7.6 (3.5)	9.5 (2.8)	0.84	[0.81, 0.88]	<.001
Irregular grammar at 24 months^g^		5,750	13.1 (12.4)	19.5 (13.8)	0.96	[0.95, 0.97]	<.001
Denver Communication Scale at 18 months^g^		5,775	−0.33 (1.10)	0.04 (0.98)	0.68	[0.59, 0.78]	<.001
Early literacy and learning							
School entry assessment (age 4–5 years): reading^d^	Achieved level	4,633	54 (7.2)	698 (92.8)	1.00		<.001
	Above expectations		130 (3.3)	3,751 (96.7)	0.45	[0.32, 0.62]	
School entry assessment (age 4–5 years): writing^d^	Achieved level	4,634	76 (6.1)	1,178 (93.9)	1.00		<.001
	Above expectations		108 (3.2)	3,272 (96.8)	0.51	[0.38, 0.69]	
Other early developmental variables							
ALSPAC developmental scale fine motor at 6 months^g^		5,537	−0.18 (1.05)	−0.00 (0.98)	0.83	[0.73, 0.96]	.009
ALSPAC developmental scale social score at 18 months^g^		5,786	−0.17 (1.11)	0.01 (0.98)	0.83	[0.72, 0.95]	.005
ALSPAC developmental scale fine motor score at 18 months^g^		5,757	−0.11 (1.11)	0.05 (0.96)	0.86	[0.75, 0.98]	.023
ALSPAC developmental scale gross motor at 18 months^g^		5,783	−0.22 (1.29)	−0.00 (0.94)	0.82	[0.73, 0.92]	.002
ALSPAC developmental scale social score at 30 months^g^		5,142	−0.26 (1.05)	0.02 (0.98)	0.76	[0.66, 0.87]	<.001
ALSPAC developmental scale fine motor score at 30 months^g^		5,121	−0.14 (1.06)	0.06 (0.97)	0.82	[0.72, 0.94]	.004
ALSPAC developmental scale gross motor score at 30 months^g^		5,132	−0.32 (1.26)	−0.01 (0.96)	0.76	[0.67, 0.86]	<.001
ALSPAC developmental scale social score at 42 months^g^		5,328	−0.24 (1.22)	0.04 (0.95)	0.76	[0.67, 0.87]	<.001
ALSPAC developmental scale fine motor score at 42 months^g^		5,332	−0.32 (1.13)	0.06 (0.97)	0.71	[0.63, 0.80]	<.001
ALSPAC developmental scale gross motor score at 42 months^g^		5,335	−0.41 (1.26)	0.01 (0.96)	0.70	[0.62, 0.78]	<.001
School entry assessment (age 4–5 years): large motor^d^	Achieved level	1,549	27 (7.9)	313 (92.1)	1.00		
	Above expectations		40 (3.3)	1,169 (96.7)	0.40	[0.24, 0.66]	<.001
Laterality at 42 months^d^	Right	5,700	138 (3.6)	3,665 (96.4)	1.00		.069
	Mixed/left		88 (4.6)	1,809 (95.4)	1.29	[0.98, 1.70]	
Weak sucking at 4 weeks^d^	No	6,158	179 (3.5)	4,879 (96.5)	1.00		.009
	Yes		58 (5.3)	1,042 (94.7)	1.52	[1.12, 2.06]	
Dribbling at 4 weeks^d^	No	6,158	107 (4.7)	2,194 (95.4)	1.00		.013
	Yes		130 (3.4)	3,727 (96.6)	0.72	[0.55, 0.93]	
Drinking too fast at 4 weeks^d^	No	6,158	49 (5.0)	930 (95.0)	1.00		.048
	Yes		188 (3.6)	4,991 (96.4)	0.71	[0.52, 0.99]	
Difficulties feeding^d^	No	6,127	195 (3.6)	5,184 (96.4)	1.00		
	Yes		37 (4.9)	711 (95.1)	1.38	[0.97, 1.98]	.088

*Note.* ALSPAC = Avon Longitudinal Study of Parents and Children.

a This column shows how the variables were grouped in the second stage within-group multivariable analysis.

b For categorical variables only.

c Where the variable of interest is categorical, the two numbers refer to *n* (%), where % is the percentage within that case/control group. The reference category for each variable can be identified by its odds ratio of 1.00. Where the variable of interest is continuous, the two numbers refer to *M* (*SD*), and the odds ratio relates to the change in odds for a one-unit increase in the exposure variable. The exceptions to this are the odds ratio for IQ and MacArthur scores, which are based on a change of 10 units.

d Categorical variable.

e “O level” was the qualification obtained at age 16 years when the parents of the cohort were at school.

f Supplemented with father's social class when the mother's occupation was not available.

g Continuous variable.

h People per room.

**Table 9.** Within-group and final between-groups multivariable regression models for early childhood risk factor variables associated with case status.

Variable	Category^a^	Within-group multivariable model		*p* value	Between-groups final multivariable model		*p* value
		Odds ratio (*n* = sample size)	95% confidence intervals		Odds ratio	95% confidence intervals	
Demographics		*n* = 5,796			*n* = 5,066		
Gender^b^	Female	1.00		<.001	1.00		.170
	Male	2.12	[1.59, 2.82]		1.25	[0.91, 1.73]	
Maternal occupation^b^^,^ ^c^	Nonmanual	1.00		.014			
	Manual	1.50	[1.09, 2.06]				
Home ownership^b^	Mortgaged/owned	1.00		<.001	1.00		.036
	Rented/other	1.85	[1.33, 2.57]		1.52	[1.04, 2.23]	
Language environment		*n* = 5,652					
Mother reads to child at 18 months^b^	Almost daily	1.00		.021	NA		
	Three to five times per week	1.34	[0.95, 1.87]				
	Less than three times per week	1.74	[1.15, 2.63]				
Overcrowding index at 8 weeks of gestation^b^	≤ 0.50	1.00		.002			
	0.50–0.75	1.41	[1.03, 1.94]		NA		
	0.75–1.00	1.37	[0.92, 2.04]				
	> 1.00	2.90	[1.69, 4.97]				
Family history of speech and language therapy attendance^b^	No	1.00		.006	NA		
	Yes	2.11	[1.29, 3.45]				
Early speech and language performance		*n* = 5,246					
Intelligibility to others at 38 months^b^	Mostly	1.00		<.001	1.00		<.001
	Sometimes/rarely	2.47	[1.74, 3.50]		2.38	[1.66, 3.40]	
Word combination at 24 months^b^	Often	1.00		.005	1.00		.006
	Sometimes	1.76	[1.21, 2.56]		1.81	[1.23, 2.67]	
	Not yet	1.83	[1.18, 2.84]		1.81	[1.15, 2.86]	
Word morphology at 38 months^d^		0.91	[0.86, 0.96]	<.001	0.91	[0.86, 0.96]	.001
Early literacy and learning		*n* = 4,633					
School entry assessment (age 4–5 years): reading^b^		0.56	[0.39, 0.81]	.002	NA		
School entry assessment (age 4–5 years): writing^b^		0.64	[0.45, 0.89]	.010	NA		
Other early developmental variables		*n* = 5,220					
ALSPAC developmental scale fine motor score at 42 months^d^		0.81	[0.70, 0.94]	.005	NA		
ALSPAC developmental scale gross motor score at 42 months^d^		0.77	[0.67, 0.88]	<.001	NA		
Weak sucking at 4 weeks^b^		1.58	[1.13, 2.20]	.009	1.45	[1.01, 2.09]	.050
Dribbling at 4 weeks^b^		0.69	[0.52, 0.92]	.012	NA		

*Note.* NA = not applicable, as the *p* value at this stage of the analysis was above the threshold of .5; ALSPAC = Avon Longitudinal Study of Parents and Children.

a For categorical variables only.

b Categorical variable.

c Supplemented with father's social class when the mother's occupation was not available.

d Continuous variable.

Maternal social class was excluded because of its association with home ownership (*p* < .001). Although both were to some extent independently associated with the outcome in the relevant within-group model, they are likely to confound each other in later models. Hence, only the measure with the stronger evidence was retained in the models presented here.

Table 9 also shows the results of the final between-groups multivariable regression analyses. Gender was retained as an important covariate given the higher prevalence rating for boys, even though its association was no longer significant (*p* = .17). Five variables were independently associated with case status. Case children were more likely to come from families who did not own their own homes (*p* = .036), to be less intelligible to others at 38 months (*p* < .001), to use single words rather than two- or three-word phrases at 24 months (*p* = .006), to use incorrect word morphology at 38 months (*p* = .001), and to have had a weak suck as a baby (*p* = .05).

Of these variables, the strongest association was low intelligibility to strangers at 38 months (OR = 2.38). Children who used single words rather than combining words at 24 months were nearly twice as likely to be case children (OR = 1.81), whereas those with higher scores on the word morphology task at 38 months (OR = 0.91) were less likely to be case children. Being part of a family who did not own their own home and having a weak suck at age 4 weeks was associated with ORs of 1.50 and 1.45, respectively.

### School-Age Predictors

Factors with *p* values greater than .10 following univariable analysis included ethnicity, maternal age, and the DDK tasks requiring repetition of /pә/ and /kә/. These factors were excluded from further analysis. Table 10 provides descriptive statistics and univariable regression models comparing children with persistent SSD against the rest of the cohort for variables with the designated strength of evidence (*p* < .10) of an initial association. Thirteen variables showed some evidence of association (*p* < .05) with case status following the within-group multivariable analyses (see Table 11). These 13 variables were taken forward to the final stage of modeling across all groups of variables.

**Table 10.** Descriptive statistics and univariable regression model results for demographic and school-age risk factor variables associated with persistent speech sound disorder, where *p* < .10 (with maternal age and gender included regardless of their *p* values).

Grouped variable^a^	Category	Data available for each variable (*N*)	Total sample (*N*)	Case children summary data^b^	Rest of cohort summary data^b^	Univariable model		*p* value
						Odds ratio	95% confidence interval	
Demographics								
Gender^c^	Female	3,397	6,662	94 (2.8)	3,303 (97.2)	1.00		<.001
	Male	3,265		169 (5.2)	3,096 (94.8)	1.92	[1.48, 2.48]	
Level of maternal education^c^^,^ ^d^	O level	2,172	6,166	79 (3.6)	2,093 (96.4)	1.00		.025
	< O level	1,335		70 (5.2)	1,265 (94.8)	1.47	[1.05, 2.04]	
	> O level	2,659		93 (3.5)	2,566 (96.5)	0.96	[0.71, 1.30]	
Maternal occupation^c^^,^ ^e^	Nonmanual	4,772	5,909	156 (3.3)	4,616 (96.7)	1.00		<.001
	Manual	1,137		64 (5.6)	1,073 (94.4)	1.76	[1.31, 2.38]	
Home ownership^c^	Mortgaged/owned	5,181	6,199	174 (3.4)	5,007 (96.6)	1.00		<.001
	Rented/other	1,018		70 (6.9)	948 (93.1)	2.12	[1.60, 2.83]	
Maternal age^f^		6,382	6,382	29.2 (4.8)	29.1 (4.6)	1.01	[0.98, 1.03]	.720
Concurrent speech and language performance								
Language comprehension^f^	Number correct	6,655	6,655	7.2 (2.3)	7.4 (1.9)	0.95	[0.89, 1.01]	.094
DDK tasks: te^c^	Correct	5,617	6,617	209 (3.7)	5,408 (96.3)	1.00		
	Incorrect	1,002		49 (4.9)	953 (95.1)	1.33	[0.97, 1.83]	.088
DDK tasks: peteke^c^	Correct	1,581	6,616	47 (3.0)	1,534 (97.0)	1.00		
	Incorrect	5,035		212 (4.2)	4,823 (95.8)	1.43	[1.04, 1.98]	.023
DDK tasks: bedege^c^	Correct	973	6,619	26 (2.7)	947 (97.3)	1.00		
	Incorrect	5,646		233 (4.1)	5,413 (95.9)	1.57	[1.04, 2.37]	.023
Phoneme deletion^f^	Number correct	5,998	5,998	16.0 (10.1)	20.5 (9.3)	0.95	[0.94, 0.96]	<.001
Difficulty pronouncing sounds^c^	No	4,773	5,432	111 (2.3)	4,662 (97.7)	1.00		
	Yes	659		95 (14.4)	564 (85.6)	7.07	[5.31, 9.43]	<.001
Nonword repetition^f^		6,640	6,640	5.7 (3.0)	7.3 (2.5)	0.78	[0.75, 0.82]	<.001
Concurrent literacy and learning								
Reading test^f^	Number correct	6,006	6,006	23.8 (10.7)	28.8 (9.0)	0.94	[0.93, 0.96]	<.001
Spelling test^f^	Combined score	5,945	5,945	20.9 (13.7)	26.3 (12.5)	0.97	[0.96, 0.98]	<.001
School assessment: reading^c^	Achieved expected level	2,842	5,644	111 (3.9)	2,731 (96.1)	1.00		
	Underachieved	513		50 (9.8)	463 (90.3)	2.66	[1.88, 3.76]	<.001
	Exceeded expected level	2,289		65 (2.8)	2,224 (97.2)	0.72	[0.53, 0.98]	
School assessment: writing^c^	Achieved expected score	4,502	5,640	153 (3.4)	4,349 (96.6)	1.00		
	Underachieved	536		60 (11.2)	476 (88.8)	3.58	[2.62, 4.90]	<.001
	Exceeded expected level	602		13 (2.3)	589 (97.8)	0.63	[0.35, 1.11]	
Identified learning problems^c^	No	4,948	5,434	162 (3.3)	4,786 (96.7)	1.00		
	Yes	486		50 (10.3)	436 (89.7)	3.39	[2.43, 4.72]	<.001
Other developmental variables (concurrent)								
Verbal IQ^f^	Number correct	6,576	6,576	10.1 (1.9)	10.7 (1.7)	0.80	[0.74, 0.86]	<.001
Performance IQ^f^	Number correct	6,567	6,567	9.4 (1.9)	10.0 (1.7)	0.81	[0.76, 0.88]	<.001
Combined IQ score^f^	Number correct	6,548	6,548	9.8 (1.9)	10.4 (1.6)	0.78	[0.72, 0.84]	<.001
Auditory memory^f^	Number correct	6,425	6,425	9.2 (3.4)	10.4 (3.1)	0.88	[0.84, 0.92]	<.001
Spatial ability^f^	Number correct	6,560	6,560	10.1 (4.3)	10.6 (3.8)	0.96	[0.93, 1.00]	.030
Attention^f^	Time taken	6,390	6,390	5.6 (2.4)	5.2 (1.9)	1.08	[1.03, 1.13]	.004
Friendships^f^	Combined score	6,360	6,360	3.8 (2.8)	3.4 (2.4)	1.05	[1.00, 1.11]	.037
Suspected coordination problem^c^	No	5,400	5,710	184 (3.4)	5,216 (96.6)	1.00		
	Yes	310		32 (10.3)	278 (89.7)	3.26	[2.20, 4.84]	<.001
Tympanostomy tubes fitted at any time^c^	No	6,020	6,404	208 (3.5)	5,812 (96.5)	1.00		
	Yes	384		41 (10.7)	343 (89.3)	3.34	[2.35, 4.75]	<.001
Hearing impairment^c^	No	5,235	5,666	178 (3.4)	5,057 (96.6)	1.00		
	Yes	431		28 (6.5)	403 (93.5)	1.97	[1.31, 2.98]	.003

*Note.* DDK = diadochokinetic.

a This column shows how the variables were grouped in the second stage within-group multivariable analysis.

b Where the variable of interest is categorical, the two numbers refer to *n* (%), where % is the percentage within that case/control group. The reference category for each variable can be identified by its odds ratio of 1.00. Where the variable of interest is continuous, the numbers are *M* (*SD*), and the odds ratio relates to the change in odds for a one-unit increase in the exposure variable. The exception to this is the odds ratio for IQ, which is based on a change of 10 units.

c Categorical variable.

d “O level” was the qualification obtained at age 16 years when the parents of the cohort were at school.

e Supplemented with father's social class when the mother's occupation was not available.

f Continuous variable.

**Table 11.** Within-group and final between-groups multivariable regression models for school-age risk factor variables associated with case status.

Variable	Category	Within-group multivariable model		*p* value	Between-groups final multivariable model		*p* value
		Odds ratio (*n* = sample size)	95% confidence intervals		Odds ratio	95% confidence intervals	
Demographics		*n* = 5,796			*n* = 4,303		
Gender^a^	Female	1.00		<.001	1.00		.003
	Male	2.12	[1.59, 2.82]		1.69	[1.18, 2.42]	
Maternal occupation^a^^,^ ^b^	Nonmanual	1.00		.014	NA		
	Manual	1.50	[1.09, 2.06]				
Home ownership^a^	Mortgaged/owned	1.00		<.001	1.00		.028
	Rented/other	1.85	[1.33, 2.57]		1.64	[1.07, 2.50]	
Speech and language performance (concurrent)		*n* = 5,415					
Difficulty pronouncing certain sounds^a^	No	1.00		<.001	1.00		<.001
	Yes	6.21	[4.63, 8.33]		5.59	[3.94, 7.94]	
Nonword repetition^c^	Number correct	0.82	[0.78, 0.87]	<.001	0.82	[0.76, 0.87]	<.001
Literacy and learning (concurrent)		*n* = 4,238					
Reading test^c^	Number correct	0.98	[0.96, 1.00]	.049	NA		
School assessment: writing^a^	Achieved expected level	1.00		.027	NA		
	Underachieved	2.04	[1.22, 3.41]				
	Beyond expected	0.85	[0.44, 1.66]				
Identified learning problems^a^	No	1.00		.003	NA		
	Yes	2.03	[1.29, 3.20]				
Other developmental variables (concurrent)		*n* = 4,802					
Combined IQ score^c^	Number correct	0.88	[0.80, 0.98]	.016	NA		
Auditory memory^c^	Number correct	0.93	[0.88, 0.98]	.006	NA		
Spatial ability^c^	Number correct	NA			NA		
Attention^c^	Number correct	NA			NA		
Friendships^c^	Score	NA			NA		
Suspected coordination problem^a^	No	1.00		<.001	1.00		.011
	Yes	2.45	[1.52, 3.95]		2.05	[1.21, 3.46]	
Tympanostomy tubes fitted at any time^a^	No	1.00		<.001	1.00		.005
	Yes	2.36	[1.48, 3.77]		2.18	[1.30, 3.64]	
Hearing impairment^a^	No	1.00		.023	1.00		.017
	Yes	1.76	[1.11, 2.80]		1.94	[1.16, 3.24]	

*Note.* NA = not applicable, as the *p* value at this stage of the analysis was above the threshold of .5.

a Categorical variable.

b Supplemented with the father's social class when the mother's occupation was not available.

c Continuous variable.

Table 11 shows that in the final model four variables remained strongly associated with case status: reported difficulty pronouncing certain sounds and nonword repetition (*p* < .001), gender (*p* = .003), and tube insertion (*p* = .005). There was weaker evidence for three further variables: home ownership (*p* = .028), suspected coordination problem (*p* = .011), and hearing impairment (*p* = .017). The strongest association was for reported difficulty pronouncing certain sounds (OR = 5.6). Children who had tube insertion and/or hearing impairment and those for whom coordination problems were suspected were roughly twice as likely to be within the persistent SSD case group (ORs of approximately 2), whereas higher scores on the nonword repetition task were associated with a decreased risk of being in the case group (OR = 0.82). In terms of demographic factors, case children were more likely to be boys and from families who did not own their own homes (ORs of approximately 1.6).

## Discussion

Using prospectively collected data from a large population-based cohort, we obtained a prevalence estimate of 3.6% for persistent SSD at 8 years. Children with persistent SSD in this study were more likely to be boys and to be from families who do not own their own homes. Early childhood predictors associated with persistent SSD were lower SES, low intelligibility to strangers at 38 months, early speech and language delay, and weak sucking as a baby. School-age predictors associated with persistent SSD were hearing impairment (>20 dB loss) on assessment at age 7 years, a history of tympanostomy tube insertion, parental report of difficulty pronouncing sounds at age 7 years, poor performance on nonword repetition tasks, and reports of suspected motor coordination problems.

### Limitations

As with any study of this size that takes place over an extended period of time, retention of participants and missing data are a problem, and bias in the samples appears. Children attending the 8-year clinic had older mothers with higher levels of education and were more likely to be living in owner-occupied housing compared with children who did not attend. However, good coverage across all levels of education and SES was maintained in the sample.

The control group was limited to just 50 participants; three of these participants were identified as outliers on the basis of their PCC-A and PCC late 8 scores relative to the rest of the control group. Time and funding considerations prevented the transcription of a greater number of control samples, but without doubt this would have added weight to the analysis. The three outliers constitute 6% of the control sample, which is nearly twice the size of the prevalence estimate obtained from this data set. It is impossible to know whether the three identified outliers represent exceptional data or whether the rest of the cohort, which functioned as a control group for the identification of predictor variables, was in fact more varied than has been assumed. For the purposes of this article it has been assumed that three outliers do indeed constitute exceptional data; however, without the benefit of further transcribed samples from the rest of the cohort, this cannot be confirmed. The findings from this article must therefore be interpreted bearing this in mind.

Information on comorbidities was patchy and therefore unreliable, making it impossible to determine the extent to which a child's presenting SSD was part of a more general learning or developmental disorder or linked to a diagnosis of childhood apraxia of speech or other neurological or structural condition. Given this limitation, the study has focused on reporting the results of the large heterogeneous group of children who could be described as having persistent SSD and has not tried to link findings to etiology or to identify subgroups in terms of risk factors. Although it was possible to build language scores into the regression analysis to allow some consideration of the level of comorbidity with language deficits, an analysis has not been carried out to determine to what extent comorbid language impairment can explain the findings. Previous work by the authors using a subset of the data presented here combined with longitudinal findings found that expressive language skill at ages 2 and 5 years was predictive of speech outcome at age 8 years (Roulstone et al., 2009), suggesting that many children in the sample described in this article may have had additional language problems. This is consistent with the findings of Shriberg et al. (1999), who found that almost half of their sample of children with SSD also had language impairment. Many previous studies (Reilly et al., 2010; Stanton-Chapman et al., 2002; B. Tomblin, Smith, & Zhang, 1997; Zubrick et al., 2007) have looked at risk factors for language impairment in younger children. A future investigation that considers the relative importance of speech factors compared with language factors in older children in this data set would be invaluable in understanding which variables explain both speech and language problems and which are exclusive to one or the other.

The benefits of using the large ALSPAC data set are offset by the limitations involved in collecting and analyzing the information in such a large cohort. This affects the level of detail available for some variables. For example, data on the family history of speech and language impairment or interventions received rely on single questions requiring parental recall. However, concurrent evidence detailing intervention suggests that a very low dosage of therapy was typical (Glogowska, Roulstone, Enderby, & Peters, 2000). There were also limitations with the variables relating to hearing. Information on the dates of tube insertion was not available, and pure-tone audiometry results were available for the children only at age 7 years and not at the same time as the speech assessment.

Last, it was not possible to complete the reliability of the transcribed samples until later in the study, meaning that discrepancies were uncovered after it was possible to resolve them. However, a reliability figure of 93% is comparable to that of other studies of typical and disordered speech (Shriberg et al., 1999). The study started with a large number of variables; therefore, although the analyses would have attended to a wide variety of potential confounding effects, the results of all such models should be considered exploratory—at least until replicated elsewhere.

### Case Identification

Identification of the case group required making a distinction between what constitutes pathology and what reasonably can be considered “typical” behavior. The wide range of variation in typical development of speech and language and the continuum from typical to atypical speech makes this process difficult, and the range of definitions used in the literature seems to confirm that there is no easy solution to this dilemma. The context of this study (a large population-based sample) allowed the identification of a case group in comparison to immediate peers rather than the need to use normative data from very different samples. However, the overlap between the scores of children with observed errors and the scores of the 50 children drawn from the rest of the cohort shows that it is still challenging to identify distinct case and noncase groups.

Some might believe that the term *persistent SSD* should include children with common clinical distortions. However, for the purpose of this study, we opted for a narrower definition of persistent SSD that is based on the fact that children in the United Kingdom whose speech errors are restricted to common clinical distortions are excluded from access to services.

Nevertheless, it is important to acknowledge that the common clinical distortions group may include children who would have fulfilled criteria for SSD at a younger age and may share some characteristics with those children defined in this study as having persistent SSD. Although there is a need to look at trajectories within the ALSPAC sample to determine case status over time, a separate study has considered how the common clinical distortions group compared against the persistent SSD group and those who did not reach criteria for case status in terms of gender, SES, IQ, nonword repetition, and DDK tasks (Wren et al., 2012). Although the case group and those who did not reach criteria for case status shared similar characteristics and were different from the common clinical distortions group on most measures, the common clinical distortions group was more similar to the persistent SSD group on measures of DDK, suggesting that there may be some overlap in their areas of difficulty in the area of rapid speech movements.

### Prevalence

The prevalence of 3.6% obtained in this study for persistent SSD is consistent with findings from other studies carried out in other English-speaking countries (Kirkpatrick & Ward, 1984: 4.6% of children aged 5–7 years in Australia; Shriberg et al., 1999: 3.8% of children aged 6 years in the United States). However, there are important differences in how the numbers are derived. Single word naming (Kirkpatrick & Ward, 1984) provides a rapid means of case identification but may miss errors that occur across word boundaries (Howard, 2004, 2007) and that would be observed in the connected speech samples used in this study and that of Shriberg et al. (1999).

This study and that of Shriberg et al. share other characteristics (i.e., children with concomitant language impairment and motor disorder were included in the sample) but differ in the way that cases were identified. Shriberg et al.'s figures are based on a multiple categorical system from the Speech Disorders Classification System in which a range of possible classifications of speech status are available. Prevalence was calculated for the specific category of speech delay, which is based on the presence of substitution or deletion errors for four or more consonants or for two or three consonants and vowels (Shriberg et al., 1997b). In contrast, this study used a cutoff point on two measures of PCC compared with a control group of children. Although it is anticipated that the two case groups are broadly similar, some differences in the composition of each group are likely to exist.

In other studies that also used direct assessment of children's speech, higher prevalence figures of 16.5% (Jessup et al., 2008) and 8.7% (Tuomi & Ivanoff, 1977) for children aged 6 years were obtained. These studies used a more tolerant definition of case status, including children with milder problems. If children with common clinical distortions only (7.88% of the sample) had been included in the persistent SSD group here, prevalence would have reached 11.4%—a more comparable figure.

Nevertheless, the prevalence figure of 3.6% for persistent SSD alone is a robust estimate of clinical need. It was obtained from a large population study and has been defined with clear parameters. It suggests that in a class of 30 school children aged 8 years, there is likely to be one child with a clinically significant speech problem.

### Predictor Variables Associated With Persistent SSD

Analysis of the predictor variables using a staged multivariable regression approach led to the identification of a small number of important variables within the two broad categories of early childhood and school-age predictors on the basis of the age of the child when the variable was measured. Results are discussed below across the categories of early childhood and school-age predictors but within the subcategories of demographics, family and environment, speech and language performance, literacy and learning skills, and other developmental measures.

#### Demographics

Low SES, as measured by home ownership, was an important predictor of persistent SSD for both the early childhood and school-age categories, whereas male gender was an important variable in the school-age predictors only. With regard to SES, reports in the literature have been conflicting, with some providing support for a relationship with SSD (Eadie et al., 2015; Shriberg et al., 1999; Winitz & Darley, 1980) and others not (Keating et al., 2001; McKinnon et al., 2007). Variation in how SES is measured may account for these differences, whereas Law et al. (2000) pointed to the possibility that SES could be operating as a proxy variable in some instances and should therefore be treated with caution.

In this study, maternal education and occupation were also included as measures of SES in the analysis, but only home ownership remained in the final model. This contrasts with Campbell et al.'s (2003) study of risk factors for SSD in 3-year-olds, which found that of two measures of SES—maternal education and health insurance categories—maternal education was more important. For this study, this raises the question of whether low SES was an important factor in accounting for variance in the findings or whether another factor (or factors) related to home ownership was associated with persistent SSD. Factors commonly linked to living in rented accommodations (e.g., lack of stability and financial security, suitability and size of living area, and quality of accommodations) could affect family interactions and thus speech development over and above low SES in isolation.

As in other prevalence studies, a greater number of boys than girls were identified with persistent SSD in this sample (Campbell et al., 2003; Eadie et al., 2015; Harrison & McLeod, 2010). Although gender was important in the school-age predictor group, the results from the early childhood predictors analysis suggest that when considered alongside other factors, it is not as important as variables relating to the environment and early development. This is consistent with the findings of Fox et al. (2002), who found gender to be less important than family history, pre- and perinatal history, and the use of pacifiers.

#### Family and Environment

None of the variables relating to family and environmental factors remained in the final model. Although some variables showed evidence at the within-group multivariable stage (mother reading to the child daily at 18 months, overcrowding at 8 weeks prenatally, and family history), they did not remain in the model after adjustment for the other variables in the between-groups multivariable analysis. This contrasts with Harrison and McLeod's (2010) study of 4,983 children, which found that parity (older siblings) was a risk factor and that use of other languages by parents was protective. However, case status in the Harrison and McLeod study was determined by parental report of concern rather than direct assessment and analysis of speech, as in this study. This method of classification achieved a positive response from 25.2% of the sample, suggesting a much larger and more diverse case group than the 3.6% identified in this study.

Although there is contradictory evidence in the literature for many of these factors, the most surprising finding is that family history did not remain in the final model as an important predictor. This factor has emerged as an important predictor in a number of studies of SSD specifically and speech and language impairment more generally (Campbell et al., 2003; Eadie et al., 2015; Felsenfeld & Plomin, 1997; Fox et al., 2002; Lewis et al., 2006, 2007; J. B. Tomblin et al., 1991). However, in this study family history was measured by a single questionnaire item regarding referral to specialist services, thus relying on parents' ability to recall information from their own early childhood. By contrast, other studies used more comprehensive questionnaires devoted specifically to the issue of family history of speech and language difficulties (Campbell et al., 2003; Felsenfeld & Plomin, 1997; J. B. Tomblin et al., 1991), used interviews and direct testing as part of a genetic linkage study (Lewis et al., 2006, 2007), or asked about whether the parents had experienced problems with speech and language in childhood rather than about referral to specialist services (Fox et al., 2002).

Other environmental factors that have been associated with SSD generally but were not identified as being independently associated with persistent SSD in this study included factors relating to birth. In the literature there are mixed findings (Campbell et al., 2003; Fox et al., 2002), and although the findings from this study suggest that pregnancy complications and smoking during pregnancy were not important, the measure used to account for this was crude. It is possible that more sensitive measures may produce associations with specific aspects related to pregnancy and birth. Overcrowding; family size; attendance at a playgroup, nursery, or childminder; and reading to the child were not associated with persistent SSD at age 8 years once other factors had been taken into account.

#### Speech and Language Performance

Difficulty pronouncing sounds, as measured via parent questionnaire when the children were aged 7 years, was the strongest predictor variable. This is not surprising given the method of identification of persistent SSD. The second strongest predictor was difficulty being understood by nonfamily members at age 38 months. For many children in the persistent SSD group presentation at age 8 years reflects a speech sound system that was typically immature when they were younger, so the fact that they presented with unintelligible speech at a younger age is not remarkable. Further research is needed to determine the degree to which this association is consistent over time and to what extent it identifies children with SSD at younger ages as well as the persistent group. If an association is not found for children with transient SSD when younger, then this could act as a useful clinical marker for persistent SSD.

Strong associations were also observed between persistent SSD and combining words at age 24 months and use of word morphology at 38 months as reported by the mother. Combining words was important at age 24 months but not at age 38 months, suggesting that this risk factor is age dependent. Highman et al. (2008) also found word combinations to be a predictor of later speech status—in their case, childhood apraxia of speech as well as difficulties with gross motor development, feeding, and dribbling. This suggests a possible motor component to this delay. It is possible that children at risk of persistent SSD may have greater difficulty in making the sequenced fine movements of the articulators required for speech and have particular trouble making the transition to word combinations, where even greater coordination of movement is required. The evidence from the other developmental school-age predictors discussed below, in which there was an association between suspected coordination problems and persistent SSD status, provides further support for this idea.

Correct use of word morphology at 38 months was based on parental report of 12 items. The OR reported showed that children scoring higher on this were less likely to be case children. Whether this reflects a language difficulty per se or a difficulty in expressing word morphology due to restrictions in speech production is not clear from these results and needs further investigation.

The results from this study suggest an association between poor performance on nonword repetition and persistent SSD status. Nonword repetition is well recognized as a measure of phonological working memory (Gathercole, Willis, Baddeley, & Emslie, 1994); it has been associated with measures of vocabulary development in typically developing children and with poor performance in children with language impairment (for a review see Coady & Evans, 2008). However, nonword repetition requires a number of processing skills beyond that of memory, including speech perception and discrimination, phonological encoding, phonological assembly, motor planning, and articulation—skills that are associated more typically with speech processing and production than with language (Stackhouse & Wells, 1997). Indeed, a recent investigation by Farquharson (2015) found that children aged between 9 and 13 years with remediated SSD performed significantly worse on a test of nonword repetition. It is not yet clear to what extent language skills may also influence the associations observed.

The remaining variables in this group (Wechsler Objective Language Dimensions comprehension, DDK accuracy, and phoneme deletion) were important at the univariable stage but were lost when considered alongside difficulty pronouncing sounds and nonword repetition. Dropping such factors from the model does not necessarily imply that they do not reflect important underlying characteristics; rather, it implies that there is shared variance among certain variables (e.g., nonword repetition and phoneme deletion) and that influences are better represented by other (more statistically dominant) measures.

#### Literacy and Learning Performance

Variables relating to literacy and learning performance did not remain in the final model in either the early childhood or school-age predictors groups when factors unrelated to literacy and learning were included. This suggests that although there is an association between literacy and learning and persistent SSD, there is considerable shared variance with other variables in the model that emerge as more statistically dominant in the analysis.

#### Other Early Developmental Variables

A range of measures remained important at the within-group stage (i.e., gross and fine motor skills at 42 months, dribbling at 4 weeks, intelligence, and memory). However, only weak sucking at 4 weeks, suspected coordination problems, and variables related to hearing (presence of hearing impairment and previous insertion of tympanostomy tubes) remained in the final model.

With regard to hearing, although there is some suggestion that there may be an impact on some subtle language skills (e.g., aspects of phonological processing or verbal working memory; Majerus et al., 2005; Nittrouer & Burton, 2005), a strong body of evidence suggests that the impact of otitis media and associated hearing loss on the development of speech and language is negligible (Paradise et al., 2005, 2007; Roberts, Hunter, et al., 2004; Roberts, Rosenfeld, & Zeisel, 2004). The contrast in these findings may relate to the differences in when the measures were taken. Roberts, Rosenfeld, et al. (2004) carried out a meta-analysis of 14 studies, and Paradise and colleagues collected longitudinal data; however, the measure used in the analysis reported here was a single hearing assessment and parental report of whether tubes had been fitted. Complementary data provided by successive hearing tests over time and information on the dates and timings of tube insertion would provide a more complete picture and clearer data relating to hearing history rather than performance at a single point in time.

Oral sucking habits have been associated with SSD in other studies of risk factors for SSD (Highman et al., 2008; Tomblin et al., 1991). Moreover, evidence associates poor sucking with other developmental factors such as early growth faltering, low IQ, and delayed gross motor development (Emond, Drewett, Blair, & Emmett, 2007; Motion, Northstone, Emond, Stucke, & Golding, 2002). It is thought that sucking difficulties in the first few weeks of life may be a marker of subtle neurological impairment, accounting for the lowered IQ score, though a recent systematic review was unable to confirm this (Slattery, Morgan, & Douglas, 2012).

Links between intelligence and memory and speech development have been shown in previous studies (Keating et al., 2001; Shriberg et al., 1999). However, this study suggests that coordination skills are more important in children with persistent SSD at age 8 years. This is consistent with reports in the literature of links between general coordination problems and speech impairment (Gaines & Missiuna, 2007; Gibbon, 2002; Hill, 2001; Hill & Bishop, 1998; Robinson, 1991; Visscher et al., 2007, 2010; Webster et al., 2005).

### Nature of Persistent SSD

The pattern of predictor variables that emerge as important in this data set helps further our understanding of the nature of persistent SSD. The findings relating to motor skill, as evidenced by a number of variables, suggest that this could be a feature common to many children identified as having persistent SSD. Problems with weak sucking as a baby and suspected coordination disorder point to a more motor-based deficit of speech. Although DDK—another measure relating to oromotor skill—was not important in the final model, it was identified as a distinguishing feature in previous work using the same data set (Wren et al., 2012). In contrast, most measures of cognition did not remain in the final model. The exception to this was nonword repetition, which encompasses a wide range of skills, including memory, phonological processing, and speech motor skill. Although the findings of this study support the concept of SSD being multifactorial in nature and although the sample included in the study was undoubtedly heterogeneous, the results hint at the possibility that when SSD persists it is multifactorial in nature and that there is involvement across more than one domain of motor skills, cognition, and language.

## Conclusions

This study investigated persistent SSD in children in a population study and obtained an estimated prevalence of 3.6%. The final model of risk factors described in the article provides useful information on what factors might be important to consider in assessing an individual child's risk for persistent SSD in the clinical setting. In the early years, limited combining of words at 24 months and use of word morphology at 38 months as well as difficulty being understood by strangers at age 3 years could be useful clinical markers alongside demographic factors relating to home ownership and gender and difficulties with nonword repetition at school age.

The predictor variables also provide useful information on the nature of persistent SSD. It is known that speech development requires intact motor, cognitive, and linguistic skills. Difficulty with any one of these areas might lead to differences in the timing and pattern of SSD, and problems in more than one area may be an important factor in determining why some children's problems with speech persist. Further research is needed to investigate this hypothesis and to determine the degree to which intervention can affect these underlying skills to remediate SSD before it can be classified as persistent.

## Supplementary Material

10.1044/2015_JSLHR-S-14-0282S1 Supplemental Table 1. Description of all variables included in the first stage univariable analysis of demographic and early childhood predictor variables associated with persistent SSD

10.1044/2015_JSLHR-S-14-0282S2 Supplemental Table 2. Description of all variables included in the first stage univariable analysis of demographic and school-age predictor variables associated with persistent SSD

10.1044/2015_JSLHR-S-14-0282S3 Supplemental Table 3. Results of first stage univariable analysis and descriptive statistics for demographic and early childhood variables associated with persistent SSD

10.1044/2015_JSLHR-S-14-0282S4 Supplemental Table 4. Results of first stage univariable analysis and descriptive statistics for demographic and school-age predictor variables associated with persistent SSD

## Footnotes

1 Children in the United Kingdom start school in the September after their fourth birthday.

2 Where information on maternal occupation was unavailable, paternal occupation was used instead.

## References

[bib1] Bird J., Bishop D. V. M., & Freeman N. (1995). Phonological awareness and literacy development in children with expressive phonological impairments. Journal of Speech and Hearing Research, 38, 446–462. 7596110 10.1044/jshr.3802.446

[bib2] Bishop D. V., & Adams C. (1990). A prospective study of the relationship between specific language impairment, phonological disorders and reading retardation. Journal of Child Psychology and Psychiatry and Allied Disciplines, 31, 1027–1050. 10.1111/j.1469-7610.1990.tb00844.x 2289942

[bib3] Bishop D. V., & Edmundson A. (1987). Language-impaired 4-year-olds: Distinguishing transient from persistent impairment. Journal of Speech and Hearing Disorders, 52, 156–173. 3573746 10.1044/jshd.5202.156

[bib4] Boyd A., Golding J., MacLeod J., Lawlor D., Fraser A., Henderson J., … Davey-Smith G. (2012). Cohort profile: The “children of the 90s”—The index offspring of the Avon Longitudinal Study of Parents and Children. International Journal of Epidemiology, 42, 111–127. 22507743 10.1093/ije/dys064 PMC3600618

[bib5] Bralley R. C., & Stoudt R. J. (1977). A 5-year longitudinal study of development of articulation in elementary school children. Language, Speech, and Hearing Services in Schools, 8, 176–180.

[bib6] Broomfield J., & Dodd B. (2004). The nature of referred subtypes of primary speech disability. Child Language Teaching and Therapy, 20, 135–151.

[bib7] Browning G. G., Rovers M. M., Williamson I., Lous J., & Burton M. J. (2010). Grommets (ventilation tubes) for hearing loss associated with otitis media with effusion in children. Cochrane Database of Systematic Reviews. doi:10.1002/14651858.CD001801.pub3 10.1002/14651858.CD001801.pub3 PMC13092889 20927726

[bib8] Campbell T. F., Dollaghan C. A., Rockette H. E., Paradise J. L., Feldman H. M., Shriberg L. D., … Kurs-Lasky M. (2003). Risk factors for speech delay of unknown origin in 3-year-old children. Child Development, 74, 346–357. 12705559 10.1111/1467-8624.7402002

[bib9] Choudhury N., & Benasich A. A. (2003). A family aggregation study: The influence of family history and other risk factors on language development. Journal of Speech, Language, and Hearing Research, 46, 261–272. 10.1044/1092-4388(2003/021) PMC1569819 14700370

[bib10] Coady J. A., & Evans J. L. (2008). Uses and interpretations of non-word repetition tasks in children with and without specific language impairments (SLI). International Journal of Language & Communication Disorders, 43(1), 1–40. 10.1080/13682820601116485 PMC5524521 18176883

[bib11] Dale P. S., Price T. S., Bishop D. V. M., & Plomin R. (2003). Outcomes of early language delay: I. Predicting persistent and transient language difficulties at 3 and 4 years. Journal of Speech, Language, and Hearing Research, 46, 544–560. 10.1044/1092-4388(2003/044) 14696985

[bib12] Delgado C. E. F., Vagi S. J., & Scott K. G. (2005). Early risk factors for speech and language impairments. Exceptionality, 13, 173–191.

[bib90] Dodd B., Holm A., Hua Z., & Crosbie S. (2003). Phonological development: A normative study of British English-speaking children. Clinical Linguistics and Phonetics, 17, 617–643. 14977026 10.1080/0269920031000111348

[bib91] Eadie P., Morgan A., Ukoumunne O. C., Ttofari Eecen K., Wake M., & Reilly S. (2015). Speech sound disorder at 4 years: Prevalence, comorbidities, and predictors in a community cohort of children. Developmental Medicine & Child Neurology, 57, 578–584. 25403868 10.1111/dmcn.12635

[bib13] Emond A., Drewett R., Blair P., & Emmett P. (2007). Postnatal factors associated with failure to thrive in term infants in the Avon Longitudinal Study of Parents and Children. Archives of Disease in Childhood, 92, 115–119. 16905563 10.1136/adc.2005.091496 PMC2083322

[bib14] Farquharson K. (2015). After dismissal: Examining the language, literacy and cognitive skills of children with remediated speech sound disorders. Perspectives on School-Based Issues, 16, 50–59.

[bib15] Felsenfeld S., Broen P. A., & McGue M. (1992). A 28-year follow-up study of adults with a history of moderate phonological disorder: Linguistic and personality traits. Journal of Speech and Hearing Research, 35, 1114–1125. 1280310 10.1044/jshr.3505.1114

[bib16] Felsenfeld S., Broen P. A., & McGue M. (1994). A 28-year follow-up of adults with a history of moderate phonological disorder: Educational and occupational results. Journal of Speech and Hearing Research, 37, 1341–1353. 7877292 10.1044/jshr.3706.1341

[bib17] Felsenfeld S., & Plomin R. (1997). Epidemiological and offspring analyses of developmental speech disorders using data from the Colorado adoption project. Journal of Speech, Language, and Hearing Research, 40, 778–791. 10.1044/jslhr.4004.778 9263943

[bib92] Fenson L., Dale P., Reznick J., Thal D., Bates E., Hartung J., … Reilly J. (1993). The MacArthur Communicative Development Inventories: User's guide and technical manual. San Diego, CA: Singular Publishing.

[bib18] Fox A. V., Dodd B., & Howard D. (2002). Risk factors for speech disorders in children. International Journal of Language & Communication Disorders, 37, 117–131. 12012611 10.1080/13682820110116776

[bib19] Gaines R., & Missiuna C. (2007). Early identification: Are speech/language-impaired toddlers at increased risk for developmental coordination disorder? Child, 33, 325–332. 10.1111/j.1365-2214.2006.00677.x 17439447

[bib20] Gathercole S. E., Willis C., Baddeley A., & Emslie H. (1994). The Children's Test of Nonword Repetition: A test of phonological working memory. Memory, 2, 103–127. 7584287 10.1080/09658219408258940

[bib21] Gibbon F. (2002). Investigations in clinical phonetics and linguistics. Mahwah, NJ: Erlbaum.

[bib22] Gillon G. T., & Moriarty B. C. (2007). Childhood apraxia of speech: Children at risk for persistent reading and spelling disorder. Seminars in Speech and Language, 28, 48–57. 17340382 10.1055/s-2007-967929

[bib23] Glogowska M., Roulstone S., Enderby P., & Peters T. (2000). Randomised controlled trial of community based speech and language therapy in preschool children. British Medical Journal, 321, 923. 11030677 10.1136/bmj.321.7266.923 PMC27499

[bib24] Glogowska M., Roulstone S., Peters T. J., & Enderby P. (2006). Early speech- and language-impaired children: Linguistic, literacy, and social outcomes. Developmental Medicine & Child Neurology, 48, 489–494. 16700942 10.1017/S0012162206001046

[bib93] Goodyer I., Wright C., & Altham P. M. E. (1989). Recent friendships in anxious and depressed school age children. Psychological Medicine, 19, 165–174. 2727204 10.1017/s0033291700011119

[bib94] Goodyer I., Wright C., & Altham P. M. E. (1990). Recent achievements and adversities in anxious and depressed school age children. Journal of Child Psychology & Psychiatry, 31, 1063–1077. 2289944 10.1111/j.1469-7610.1990.tb00846.x

[bib25] Harrison L. J., & McLeod S. (2010). Risk and protective factors associated with speech and language impairment in a nationally representative sample of 4- to 5-year-old children. Journal of Speech, Language, and Hearing Research, 53, 508–529. 10.1044/1092-4388(2009/08-0086) 19786704

[bib26] Hesketh A. (2004). Early literacy achievement of children with a history of speech problems. International Journal of Language & Communication Disorders, 39, 453–468. 15691075 10.1080/13682820410001686013

[bib27] Highman C., Hennessey N., Sherwood M., & Leitao S. (2008). Retrospective parent report of early vocal behaviours in children with suspected childhood apraxia of speech (sCAS). Child Language Teaching & Therapy, 24, 285–306.

[bib28] Hill E. L. (2001). Non-specific nature of specific language impairment: A review of the literature with regard to concomitant motor impairments. International Journal of Language & Communication Disorders, 36, 149–171. 11344592 10.1080/13682820010019874

[bib29] Hill E. L., & Bishop D. V. M. (1998). A reaching test reveals weak hand preference in specific language impairment and developmental co-ordination disorder. Laterality, 3, 295–310. 15513093 10.1080/713754314

[bib30] Howard S. (2004). Connected speech processes in developmental speech impairment: Observations from an electropalatographic perspective. Clinical Linguistics & Phonetics, 18, 405–417. 15573480 10.1080/02699200410001703547

[bib31] Howard S. (2007). The interplay between articulation and prosody in children with impaired speech: Observations from electropalatographic and perceptual analysis. Advances in Speech Language Pathology, 9, 20–35.

[bib32] James D. (2001). Use of phonological processes in Australian children ages 2 to 7;11 years. Advances in Speech-Language Pathology, 3, 109–127.

[bib33] Jessup B., Ward E., Cahill L., & Heating D. (2008). Prevalence of speech and/or language impairment in preparatory students in northern Tasmania. International Journal of Speech-Language Pathology, 10, 364–377. 20840035 10.1080/17549500701871171

[bib34] Keating D., Turrell G., & Ozanne A. (2001). Childhood speech disorders: Reported prevalence, comorbidity and socioeconomic profile. Journal of Paediatrics and Child Health, 37, 431–436. 11885704 10.1046/j.1440-1754.2001.00697.x

[bib35] Kirkpatrick E., & Ward J. (1984). Prevalence of articulation errors in New South Wales primary school pupils. Australian Journal of Human Communication Disorders, 12, 55–62.

[bib36] Larrivee L. S., & Catts H. W. (1999). Early reading achievement in children with expressive phonological disorders. American Journal of Speech-Language Pathology, 8, 118–128.

[bib37] Law J., Boyle J., Harris F., Harkness A., & Nye C. (2000). Prevalence and natural history of primary speech and language delay: Findings from a systematic review of the literature. International Journal of Language & Communication Disorders, 35, 165–188. 10912250 10.1080/136828200247133

[bib38] Law J., Rush R., Schoon I., & Parsons S. (2009). Modeling developmental language difficulties from school entry into adulthood: Literacy, mental health, and employment outcomes. Journal of Speech, Language, and Hearing Research, 52, 1401–1416. 10.1044/1092-4388(2009/08-0142) 19951922

[bib39] Leitao S., Fletcher J., & Hogben J. (2000). Speech impairment and literacy difficulties: Underlying links. The Australian Educational and Developmental Psychologist, 17, 63–75.

[bib40] Lewis B. A., & Freebairn L. (1997). Subgrouping children with familial phonologic disorders. Journal of Communication Disorders, 30, 385–401. 9309530 10.1016/s0021-9924(96)00110-4

[bib41] Lewis B. A., Freebairn L. A., Hansen A. J., Miscimarra L., Iyengar S. K., & Taylor H. G. (2007). Speech and language skills of parents of children with speech sound disorders. American Journal of Speech-Language Pathology, 16, 108–118. 17456889 10.1044/1058-0360(2007/015)

[bib42] Lewis B. A., Freebairn L. A., Hansen L. A., Stein C. M., Shriberg L. D., Iyengar S. K., & Taylor H. G. (2006). Dimensions of early speech sound disorders. Journal of Communication Disorders, 39, 139–157. 16386753 10.1016/j.jcomdis.2005.11.003

[bib43] Long S. H., Fey M. E., & Channell R. W. (2006). Computerized Profiling (MS-DOS Version 9.7.0). Cleveland, OH: Case Western Reserve University.

[bib44] Majerus S., Amand P., Boniver V., Demanez J., Demanez L., & Linden M. V. D. (2005). A quantitative and qualitative assessment of verbal short-term memory and phonological processing in 8-year-olds with a history of repetitive otitis media. Journal of Communication Disorders, 38, 473–498. 15950984 10.1016/j.jcomdis.2005.04.002

[bib95] Manly T., Robertson I. H., Anderson V., & Nimmo-Smith I. (1998). The Test of Everyday Attention for Children (TEAch). Bury St Edmunds, United Kingdom: Thames Valley Test Company.

[bib45] McKinnon D. H., McLeod S., & Reilly S. (2007). The prevalence of stuttering, voice, and speech-sound disorders in primary school students in Australia. Language, Speech, and Hearing Services in Schools, 38, 5–15. 10.1044/0161-1461(2007/002) 17218532

[bib46] Motion S., Northstone K., Emond A., Stucke S., & Golding J. (2002). Early feeding problems in children with cerebral palsy: Weight and neurodevelopmental outcomes. Developmental Medicine & Child Neurology, 44, 40–43. 11811650 10.1017/s0012162201001633

[bib47] Nathan L., Stackhouse J., Goulandris N., & Snowling M. J. (2004). The development of early literacy skills among children with speech difficulties: A test of the “critical age hypothesis.” Journal of Speech, Language, and Hearing Research, 47, 377–391. 10.1044/1092-4388(2004/031) 15157138

[bib48] Nittrouer S., & Burton L. T. (2005). The role of early language experience in the development of speech perception and phonological processing abilities: Evidence from 5-year-olds with histories of otitis media with effusion and low socioeconomic status. Journal of Communication Disorders, 38, 29–63. 15475013 10.1016/j.jcomdis.2004.03.006

[bib96] Northstone K., Bonnell S., Horwood J., Bell C., Sadler S., Carmichael A., & the Focus@8 study team. (2006). Avon longitudinal study of parents and children: Focus at 8 built files documentation (No. v3a). Bristol, UK: ALSPAC.

[bib97] Northstone K., Bonnell S., Sadler S., Carmichael A., & the Focus@7 study team. (2005). Avon longitudinal study of parents and children: Focus @7 built files (No. version 3c). Bristol, UK: ALSPAC.

[bib49] Pagel Paden E. (1994). Otitis media and disordered phonologies: Some concerns and cautions. Topics in Language Disorders, 14, 72–83.

[bib50] Paradise J. L., Dollaghan C. A., Campbell T. F., Feldman H. M., Bernard B. S., Colborn D. K., … Smith C. G. (2005). Otitis media and tympanostomy tube insertion during the first three years of life: Developmental outcomes at the age of four years. Pediatrics, 112, 265–277. 10.1542/peds.112.2.265 12897272

[bib51] Paradise J. L., Feldman H. M., Campbell T. F., Dollaghan C. A., Rockette H. E., Pitcairn D. L., … Pelham W. J. (2007). Tympanostomy tubes and development outcomes at 9 to 11 years of age. New England Journal of Medicine, 356, 248–261. 17229952 10.1056/NEJMoa062980

[bib52] Patel R. R., Peters T. J., Murphy D. J. & the ALSPAC Study Team. (2005). Prenatal risk factors for caesarean section. Analyses of the ALSPAC cohort of 12944 women in England. International Journal of Epidemiology, 34, 353–367. 15659468 10.1093/ije/dyh401

[bib53] Peters T. J. (2008). Multifarious terminology: Multivariable or multivariate? Univariable or univariate? Paediatric and Perinatal Epidemiology, 22, 506. 19000286 10.1111/j.1365-3016.2008.00966.x

[bib54] Peterson R. L., Pennington B. F., Shriberg L. D., & Boada R. (2009). What influences literacy outcome in children with speech sound disorder? Journal of Speech, Language, and Hearing Research, 52, 1175–1188. 10.1044/1092-4388(2009/08-0024) PMC3608470 19403946

[bib55] Raitano N. A., Pennington B. F., Tunick R. A., Boada R., & Shriberg L. D. (2004). Pre-literacy skills of children with speech sound disorders. Journal of Child Psychology & Psychiatry, 45, 821–835. 15056313 10.1111/j.1469-7610.2004.00275.x

[bib56] Records N., & Tomblin J. B. (1994). Clinical decision making: Describing the decision rules of practicing speech-language pathologists. Journal of Speech and Hearing Research, 37, 144–156. 8170120

[bib57] Reilly S., Wake M., Bavin E. L., Prior M., Williams J., Bretherton L., … Ukoumunne O. C. (2007). Predicting language at 2 years of age: A prospective community study. Pediatrics, 120, e1441–e1449. 18055662 10.1542/peds.2007-0045

[bib58] Reilly S., Wake M., Ukoumunne O. C., Bavin E., Prior M., Cini E., … Bretherton L. (2010). Predicting language outcomes at 4 years of age: Findings from Early Language in Victoria Study. Pediatrics, 126, e1530–e1537. 21059719 10.1542/peds.2010-0254

[bib59] Rescorla L. (2002). Language and reading outcomes to age 9 in late-talking toddlers. Journal of Speech, Language, and Hearing Research, 45, 360–371. 10.1044/1092-4388(2002/028) 12003517

[bib60] Rice M. L., Taylor C. L., & Zubrick S. R. (2008). Language outcomes of 7-year-old children with or without a history of late language emergence at 24 months. Journal of Speech, Language, and Hearing Research, 51, 394–407. 10.1044/1092-4388(2008/029) 18367685

[bib61] Roberts J., Hunter L., Gravel J., Rosenfeld R., Berman S., Haggard M., … Wallace I. (2004). Otitis media, hearing loss, and language learning: Controversies and current research. Journal of Developmental and Behavioral Pediatrics, 25, 110–122. 15083134 10.1097/00004703-200404000-00007

[bib62] Roberts J., Rosenfeld R. M., & Zeisel S. A. (2004). Otitis media and speech and language: A meta-analysis of prospective studies. Pediatrics, 113, e238–e248. 14993583 10.1542/peds.113.3.e238

[bib63] Robinson R. J. (1991). Causes and associations of severe and persistent specific speech and language disorders in children. Developmental Medicine & Child Neurology, 33, 943–962. 1720749 10.1111/j.1469-8749.1991.tb14811.x

[bib98] Rosner J., & Simon D. P. (1971). The Auditory Analysis Test: An initial report. Journal of Learning Disabilities, 4, 40–48.

[bib64] Roulstone S., Law J., Rush R., Clegg J., & Peters T. J. (2011). Investigating the role of language in children's early educational outcomes (Research report DFE-RR134). London, United Kingdom: Department for Education.

[bib65] Roulstone S., Miller L. L., Wren Y., & Peters T. J. (2009). The natural history of speech impairment of 8-year-old children in the Avon Longitudinal Study of Parents and Children: Error rates at 2 and 5 years. International Journal of Speech-Language Pathology, 11, 381–391.

[bib99] Rust J., Golombok S., & Trickey G. (1993). WORD: Wechsler Objective Reading Dimensions Manual. Sidcup, UK: The Psychological Corporation.

[bib66] Rust J. (1996). Wechsler Objective Language Dimensions Manual. London, United Kingdom: The Psychological Corporation.

[bib67] Rvachew S. (2007). Phonological processing and reading in children with speech sound disorders. American Journal of Speech-Language Pathology, 16, 260–270. 17666551 10.1044/1058-0360(2007/030)

[bib68] Shriberg L. D. (1993). Four new speech and prosody-voice measures for genetics research and other studies in developmental phonological disorders. Journal of Speech and Hearing Research, 36, 105–140. 8450654 10.1044/jshr.3601.105

[bib69] Shriberg L. D., Austin D., Lewis B. A., McSweeny J. L., & Wilson D. L. (1997a). The percentage of consonants correct (PCC) metric: Extensions and reliability data. Journal of Speech, Language, and Hearing Research, 40, 708–722. 10.1044/jslhr.4004.708 9263938

[bib70] Shriberg L. D., Austin D., Lewis B. A., McSweeny J. L., & Wilson D. L. (1997b). The Speech Disorders Classification System (SDCS): Extensions and lifespan reference data. Journal of Speech, Language, and Hearing Research, 40, 723–740. 10.1044/jslhr.4004.723 9263939

[bib71] Shriberg L. D., Tomblin J. B., & McSweeny J. L. (1999). Prevalence of speech delay in 6-year-old children and comorbidity with language impairment. Journal of Speech, Language, and Hearing Research, 42, 1461–1481. 10.1044/jslhr.4206.1461 10599627

[bib100] Slattery J., Morgan A, & Douglas J. (2012). Early sucking and swallowing problems as predictors of neurodevelopmental outcome in children with neonatal brain injury: A systematic review. Developmental Medicine and Child Neurology, 54, 796–806. 22607330 10.1111/j.1469-8749.2012.04318.x

[bib72] Smit A. B. (1993a). Phonologic error distributions in the Iowa-Nebraska articulation norms project: Consonant singletons. Journal of Speech and Hearing Research, 36, 533–547. 8331911 10.1044/jshr.3603.533

[bib73] Smit A. B. (1993b). Phonologic error distributions in the Iowa-Nebraska articulation norms project: Word-initial consonant clusters. Journal of Speech and Hearing Research, 36, 931–947. 8246482 10.1044/jshr.3605.931

[bib74] Stackhouse J., & Wells B. (1997). Children's speech and literacy difficulties: A psycholinguistic framework. Chichester, United Kingdom: Whurr.

[bib75] Stanton-Chapman T. L., Chapman D. A., Bainbridge N. L., & Scott K. G. (2002). Identification of early risk factors for language impairment. Research in Developmental Disabilities, 23, 390–405. 12426008 10.1016/s0891-4222(02)00141-5

[bib101] Sutherland D., & Gillon G. T. (2005). Assessment of phonological representations in children with speech impairment. Language, Speech, and Hearing Services in Schools, 36, 294–307. 10.1044/0161-1461(2005/030) 16389702

[bib76] Sutherland D., & Gillon G. T. (2007). Development of phonological representations and phonological awareness in children with speech impairment. International Journal of Language & Communication Disorders, 42, 229–250. 17365095 10.1080/13682820600806672

[bib77] Templin M. C. (1957). Certain language skills in children; Their development and interrelationships (Monograph series no. 26). Minneapolis, MN: University of Minnesota, The Institute of Child Welfare.

[bib78] Tomblin B., Smith E., & Zhang X. (1997). Epidemiology of specific language impairment: Prenatal and perinatal risk factors. Journal of Communication Disorders, 30, 325–344. 9208366 10.1016/s0021-9924(97)00015-4

[bib79] Tomblin J. B., Hardy J. C., & Hein H. A. (1991). Predicting poor-communication status in preschool children using risk factors present at birth. Journal of Speech and Hearing Research, 34, 1096–1105. 1749241 10.1044/jshr.3405.1096

[bib80] Tuomi S., & Ivanoff P. (1977). Incidence of speech and hearing disorders among kindergarten and grade 1 children. Special Education in Canada, 51, 5–8.

[bib81] Visscher C., Houwen S., Moolenaar B., Lyons J., Scherder E. J., & Hartman E. (2010). Motor proficiency of 6- to 9-year-old children with speech and language problems. Developmental Medicine & Child Neurology, 52, e254–e258. 20804514 10.1111/j.1469-8749.2010.03774.x

[bib82] Visscher C., Houwen S., Scherder E., Moolenaar B., & Hartman E. (2007). Motor profile of children with developmental speech and language disorders. Pediatrics, 120, e158–e163. 17576781 10.1542/peds.2006-2462

[bib83] Webster R. I., Majnemer A., Platt R. W., & Shevell M. I. (2005). Motor function at school age in children with a preschool diagnosis of developmental language impairment. Journal of Pediatrics, 146, 80–85. 15644827 10.1016/j.jpeds.2004.09.005

[bib102] Wechsler D., Golombok S., & Rust J. (1992). Wechsler Intelligence Scale for Children–Third UK Edition manual. Sidcup, UK: The Psychological Corporation.

[bib84] Winitz H., & Darley F. L. (1980). Speech production. In Lassman F. M., Fisch R. O., Vetter D. L., & LaBenz E. S. (Eds.), Early correlates of speech, language and hearing (pp. 232–265). Littleton, MA: PSG Publishing.

[bib85] Wolke D., & Meyer R. (1999). Cognitive status, language attainment, and prereading skills of 6-year-old very preterm children and their peers: The Bavarian longitudinal study. Developmental Medicine & Child Neurology, 41, 94–109. 10075095 10.1017/s0012162299000201

[bib103] Wren Y. (2015). “He'll grow out of it soon—won't he?”—The characteristics of older children’s speech when they do—and don't—grow out of it. Perspectives on School-Based Issues, 16, 25–36.

[bib86] Wren Y. E., McLeod S. M., White P., Miller L. L., & Roulstone S. E. (2013). Speech characteristics of 8-year-old children with atypical speech: Findings from a prospective population study. Journal of Communication Disorders, 46, 53–69. 23102668 10.1016/j.jcomdis.2012.08.008

[bib87] Wren Y. E., Roulstone S. E., & Miller L. L. (2012). Distinguishing groups of children with persistent speech disorder: Findings from a prospective population study. Logopedics, Phoniatrics and Vocology, 37(1), 1–10. 10.3109/14015439.2011.625973 22059376

[bib88] Yliherva A., Olsén P., Mäki-Torkko E., Koiranen M., & Järvelin M. R. (2001). Linguistic and motor abilities of low-birthweight children as assessed by parents and teachers at 8 years of age. Acta Paediatrica, 90, 1440–1449. 11853344 10.1111/j.1651-2227.2001.tb01611.x

[bib89] Zubrick S. R., Taylor C. L., Rice M. L., & Slegers D. W. (2007). Late language emergence at 24 months: An epidemiological study of prevalence, predictors, and covariates. Journal of Speech, Language, and Hearing Research, 50, 1562–1592. 10.1044/1092-4388(2007/106) PMC3521638 18055773

