# Supplementary material for: Prevalence and Predictors of Persistent Speech Sound Disorder at Eight Years Old: Findings From a Population Cohort Study
Source: J Speech Lang Hear Res. 2016 Aug;59(4):647–73. doi: 10.1044/2015_JSLHR-S-14-0282 (PMC5280061; doi:10.1044/2015_JSLHR-S-14-0282)
Supplement: Supplemental Table 1. [file JSLHR-59-647-s001.pdf]

Online supplemental materials, Wren et al., “Prevalence and Predictors of Persistent Speech Sound Disorder at Eight Years Old: Findings From a Population Cohort Study,” *JSLHR*, doi:10.1044/2015_JSLHR-S-14-0282

Supplemental Table 1. Description of all variables included in the first stage univariable analysis of demographic and early childhood predictor variables associated with persistent speech sound disorder (SSD).

**Grouped variables**	**Variable**	**Method of data collection**	**Specific measure**	**Timing of data collection**
Demographic	Gender (categorical: boy/girl)	Birth records from midwife		Birth
	Ethnicity (categorical: White/non-White)	Questionnaire to mother	Q: How would you describe the ethnic group of your father?<br>*White*<br>*Black/Caribbean*<br>*Black/African*<br>*Black/other (please describe below)*<br>*Indian*<br>*Pakistani*<br>*Bangladeshi*<br>*Chinese*<br>*Any other ethnic group (please describe)*	At 32 weeks gestation
	Level of maternal education (categorical: [i]<O’level/ O’level/>O’level)	Questionnaire to mother	Q: What educational qualifications do you have? *A range of options was provided representing levels pre- and post-O’level.*	At 32 weeks gestation
	Maternal occupation (categorical: nonmanual/manual)	Questionnaire to mother (supplemented with fathers if information on maternal occupation was not available)	Q: Please describe your present or last main job? *Written text subsequently coded using OPCS (Office of Population Censuses and Surveys) job codes.*	At 32 weeks gestation
	Home ownership (categorical: mortgages or owned/rented or other)	Questionnaire to mother	Q: Is your home mortgaged, owned, rented, or other?	At 8 weeks gestation
	Maternal age at birth of child	Medical records	Date of birth	Post birth

	(continuous)			
Environment	Parity (how many previous pregnancies resulted in a live birth or still birth) (categorical: 1st child, 2nd child, 3rd or more)	Questionnaire to mother	The mother was asked how many times she had been pregnant. Separate questions asked about number of terminations and miscarriages. The score for parity was derived from the total pregnancies minus the total number of terminations and miscarriages.	At 32 weeks gestation
	Languages other than English used at home (categorical: yes/no)	Questionnaire to mother	Q: Are there any other languages apart from Spoken English used in your household, including sign language for the deaf?	Child aged 81 months
	Pre-school provision: Child attends day nursery/crèche (categorical: yes/no)	Questionnaire to mother	Q: Who regularly looks after your baby when you are not there?<br><br>*Partner, baby's grandparent, other relative, friend/neighbor, paid person outside your home (e.g., child minder), paid person in your home (e.g., nanny), baby sitter day nursery (crèche), other (please describe*)	Measure repeated when child aged 8 weeks, 8 months, 15 months, 24 months, 38 months, and 54 months
	Pre-school provision: Child attends nursery, playgroup or a childminder (categorical: yes/no)	Questionnaire to mother	Q: Does your child attend nursery, playgroup or a childminder?	Child aged 33 months and 47 months
	Reading to your child (categorical: almost daily, 3–5 times per week, less than 3 times per week)	Questionnaires to mother	Q: How often do you or your partner or another person read stories to your child or show them pictures in books?	Child aged 18 months (mom and partner); child aged 24 months (mom only)
	Reading to your child (categorical: almost daily, 1–5 times per week, less than	Questionnaires to mother	Q: How often do you or your partner or another person read stories to your child or show them pictures in books?	Child aged 42 months (mom, partner, and other person)

	once per week)			
	Overcrowding (categorical: less than 0.5 people per room, between 0.5 and 0.75 people per room, between 0.75 and 1 person per room, more than one person per room)	Questionnaires to mother	Q: How many rooms are there in your house? And Q: How many people live in your household now?<br><br>The overcrowding index was calculated by dividing the total number of people by the total number of rooms.	At 8 weeks gestation and when child aged 21 months and 33 months
	Family history (categorical: yes/no)	Questionnaires to mother and partner	Q: When you were a child, did you ever go to a speech therapist?	At 12 weeks gestation
	Premature birth (categorical: yes/no)	Medical records	Was the baby born before 37 weeks gestation?	Post birth
	Method of delivery (categorical: spontaneous, assisted, elective caesarean, emergency caesarean)	Medical records	What method of delivery was used during labor?	Post birth
	Pregnancy complications—hypertension, unexplained abdominal pain, vaginal bleeding, vomiting, any complication (categorical: yes/no)	Medical records	A range of possible types of pregnancy complication were available for consideration in the analysis but many had few or no case children in the yes category. Only those variables where each cell had a value of 7 or higher were included in the analysis.	Post birth
	Breastfeeding (categorical: never, less than 3 months, more than 3 months)	Questionnaire to mother	Q: Did you breastfeed? How old was your baby when you stopped?	Child aged 6 months
	Smoking—at any time, pre-pregnancy, during 1st trimester, during the last two	Questionnaire to mother	Q: Have you ever smoked? Did you smoke regularly at any of the following time—before pregnancy, in the first three months of	At 18 weeks gestation

	weeks (categorical: yes/no)		pregnancy, in the last two weeks?	
	First child as a teenager (categorical: yes/no)	Questionnaire to mother	Q: How old were you when you became pregnant for the very first time?	At 18 weeks gestation
	Feelings (continuous)	Questionnaires to mother	Raw scores from the depression subscale of the Edinburgh PostNatal Depression Scale (Cox, Holden, & Sagovsky, 1987) and the anxiety, depression, and somaticism subscales of the Crown–Crisp Experiential Index (Crown & Crsip, 1979) at various time points.	At 8 weeks and 32 weeks gestation and when the child was aged 8 weeks, 8 months, 21 months, 33 months
**Early speech and language performance**	MacArthur understanding and saying vocabulary (continuous)	Questionnaire to mother	Raw scores from an adaptation of the MacArthur–Bates Communication Inventory (Fenson et al, 1993). Mother was presented with vocabulary items and asked to report which of these her child understands and which they say.	Child aged 38 months
	Intelligibility to mother, family, and others (categorical: mostly/sometimes or rarely)	Questionnaire to mother	Q: Can you, your family, visitors to the house understand what your child says?	Child aged 38 months
	Use of gesture (categorical: no—never/yes but not now/yes and still does)	Questionnaire to mother	Q: Does your child prefer using gestures to get what they want?	Child aged 38 months
	Word combination (categorical: often/sometimes/not yet)	Questionnaire to mother	Q: Has your child begun to combine words yet, such as ‘nother sweet’ or ‘doggie bite’?	Child aged 24 months and 38 months
	Word morphology (continuous)	Questionnaire to mother	Q: Which of these is most like the way your child talks now: two feet/two foots? Twelve similar questions enquiring about the child’s use of similar morphological constructions were	Child aged 38 months

			asked.	
	Irregular grammar (continuous)	Questionnaire to mother	Q: Which of these does your child say or understand? Mother was presented with 5 items containing irregular plurals and 20 items containing irregular past tense.	Child aged 24 months
	Stuttering (categorical: never/sometimes/often)	Questionnaire to mother	Q: Does your child stumble or get stuck on words or repeat them many times?	Child aged 38 months
	Denver Communication Score (continuous)	Questionnaires to mother	Mother was asked a range of questions on her child’s communication skills.	Child aged 6 months and 18 months
Early literacy and learning skills	School entry assessments: reading (categorical: achieved expected level; exceeded expected level)	School entry assessments	Child graded on reading task	On entry to school at age 4–5 years
	School entry assessments: writing (categorical: achieved expected level; exceeded expected level)	School entry assessments	Child graded on writing task	On entry to school at age 4–5 years
Other early developmental	Low birth weight (categorical: less than 2500 grams/more than or equal to 2500 grams)	Medical records	Weight of baby immediately after delivery	Post birth
	Avon Longitudinal Study of Parents and Children (ALSPAC) developmental scale social, fine motor and gross motor scales (continuous)	Questionnaires to mother	Mother was asked a range of questions on her child’s social and motor skills.	Child aged 6 months,18 months, 30 months, and 42 months

	School entry assessments: large and fine motor (categorical: achieved expected level/exceeded expected level)	School entry assessments	Child graded on gross and fine motor tasks	On entry to school at age 4–5 years
	Laterality (categorical: right/mixed/left)	Questionnaire to mother	Q: With which hand does your child: throw a ball, draw, hit things, use a knife, hold his toothbrush, color in?	Child aged 42 months
	Feeding difficulties (categorical: yes/no)	Questionnaire to mother	Q: Please indicate if your baby has the following feeding behaviors:<br><br>*Weak sucking*<br>*Choking*<br>*Dribbling*<br>*Drinking too fast*<br>*Exhaustion with feeding*<br>*Slow feeding*<br>*Small quantity of feeds*<br>*Feeling unsatisfied after feeding*<br>*Refusing milk*<br>Q: Do you feel your baby is difficult to feed?	Child aged 4 weeks

References

Cox, J. L., Holden, J. M., & Sagovsky, R. (1987). Development of the Edinburgh Postnatal Depression Scale. *British Journal of Psychiatry, 150*, 782–786.
Crown, S., & Crisp, A. H. (1979). *Manual of the Crown–Crisp Experiential Index*. London, United Kingdom: Hodder & Stoughton.
Fenson, L., Dale, P. S., Reznick, J. S., Thal, D., Bates, E., Hartung, J. P., . . . Reilly, J. S. (1993). *The MacArthur–Bates Communicative Development Inventories: User’s guide and technical manual*. Baltimore, MD: Brookes.

[i] O’level was the qualification obtained at age 16 years when the parents of the cohort were at school.
