# Supplementary material for: Prevalence and Predictors of Persistent Speech Sound Disorder at Eight Years Old: Findings From a Population Cohort Study
Source: J Speech Lang Hear Res. 2016 Aug;59(4):647–73. doi: 10.1044/2015_JSLHR-S-14-0282 (PMC5280061; doi:10.1044/2015_JSLHR-S-14-0282)
Supplement: Supplemental Table 2. [file JSLHR-59-647-s002.pdf]

**Supplemental Table 2.** Description of all variables included in the first stage univariable analysis of demographic and school-age predictor variables associated with persistent speech sound disorder (SSD).

| Grouped variables | Specific variable | Method of data collection | Specific measure | Timing of data collection |
|---|---|---|---|---|
| Demographic | Gender (categorical: boy/girl) | Birth records from midwife | | Birth |
| | Ethnicity (categorical: White/non-White) | Questionnaire to mother | Q: How would you describe the ethnic group of your father?<br>*White*<br>*Black/Caribbean*<br>*Black/African*<br>*Black/other (please describe below)*<br>*Indian*<br>*Pakistani*<br>*Bangladeshi*<br>*Chinese*<br>*Any other ethnic group (please describe)* | At 32 weeks gestation |
| | Level of maternal education (categorical: [i]<O’level/ O’level/>O’level) | Questionnaire to mother | Q: What educational qualifications do you have? *A range of options was provided representing levels pre- and post-O’level.* | At 32 weeks gestation |
| | Maternal occupation (categorical: non-manual/manual) | Questionnaire to mother | Q: Please describe your present or last main job? *Written text subsequently coded using OPCS (Office of Population Censuses and Surveys) job codes.* | At 32 weeks gestation |
| | Home ownership (categorical: mortgages or owned/rented or other) | Questionnaire to mother | Q: Is your home mortgaged, owned, rented, or other? | At 8 weeks gestation |
| | Maternal age at birth of child | Midwife records | Date of birth | At recruitment to study |

| | | | | |
|---|---|---|---|---|
| | (continuous) | | | |
| Concurrent speech and language performance | Language comprehension (continuous) | Listening Comprehension subtest of Wechsler Objective Language Dimensions (WOLD; Rust, 1996), Part 2 | Raw score of correct items | Focus @ 8[ii] |
| | Diadochokinetic tasks (categorical: correct/incorrect) | Repetition of a variety of different syllables for ten seconds each (*pe, te, ke, peteke, bedege*; Northstone et al., 2006) | Correct/incorrect production of all syllables in each task | Focus @ 8 |
| | Phoneme deletion (continuous) | Auditory Analysis Test (Rosner & Simon, 1971) | Raw score of correct items | Focus @ 7 |
| | Difficulty pronouncing sounds (categorical: yes/no) | Questionnaire to mother | Q: Does your child have difficulty in pronouncing certain sounds, e.g., “th,” “ss,” “t”? | Child aged 81 months |
| | Nonword repetition (continuous) | The Children’s Test of Non-word Repetition (CNRep; adapted—12 items: 4 each of 3, 4, and 5 syllables; Gathercole & Baddeley, 1996) | Total number of nonwords repeated correctly | Focus @ 8 |
| Concurrent literacy and learning performance | Reading test (continuous) | Wechsler Objective Language Dimensions (WORD) Reading subtest (Rust et al., 1993) | Raw score of correct items | Focus @ 7 |
| | Spelling test (continuous) | SpellingTest—15 words (Northstone et al., 2005) | Spelling score (3 points for correct spelling; 2 points for incorrect spelling but correct phonetically; 1 point for just one letter wrong) | Focus @ 7 |
| | School assessment: reading (categorical: achieved expected level; | Key Stage 1 Standard Attainment Tests | Child graded on reading task and reading comprehension at 3 levels | End of Year 2 in UK primary school (child aged 6/7 years) |

| | | | | |
|---|---|---|---|---|
| | underachieved; exceeded expected level) | | | |
| | School assessment: writing (categorical: achieved expected level; underachieved; exceeded expected level) | Key Stage 1 Standard Attainment Tests | Child graded on writing task at 3 levels | End of Year 2 in UK primary school (child aged 6/7 years) |
| | Identified learning problem (categorical: yes/no) | Questionnaire to mother | Q: Has your child been identified as having any particular learning problems? | Child aged 77 months |
| Other developmental | Verbal IQ (continuous) | Wechsler Intelligence Scale for Children–Third UK Edition (WISC-III$^{UK}$; Wechsler et al., 1992)—reduced form using alternate test items | Sum of the standard scores for all verbal test items (based on doubling of raw scores) | Focus @ 8 |
| | Performance IQ (continuous) | WISC-III$^{UK}$ (Wechsler et al., 1992)—reduced form using alternate test items | Sum of the standard scores for all performance test items (based on doubling of raw scores) | Focus @ 8 |
| | Combined IQ score (continuous) | WISC-III$^{UK}$ (Wechsler et al., 1992)—reduced form using alternate test items | Sum of the standard scores for all verbal and performance test items (based on doubling of raw scores) | Focus @ 8 |
| | Auditory memory (continuous) | Digit Span (subtest of WISC-III$^{UK}$)—reduced form using alternate test items | Standard score for task (based on doubling of raw score) | Focus @ 8 |
| | Spatial ability (continuous) | Block Design (subtest of WISC-III$^{UK}$)—reduced form using alternate test items | Standard score for task (based on doubling of raw score) | Focus @ 8 |

| | | | | |
|---|---|---|---|---|
| | Attention (continuous) | Sky Search task from test of everyday attention for children (TEA-Ch; Manly et al., 1998) | The time taken for the child to identify identical pairs of pictures adjusted for motor speech | Focus @ 8 |
| | Friendships (continuous) | Friendships questionnaire (Goodyer et al., 1989, 1990) | A combined raw score based on five questions to the child about friendships: How many friends do you have? Are you happy with your friends? How often do you see your friends outside school? Do your friends understand you? Do you talk to your friends about problems? | Focus @ 8 |
| | Suspected coordination problem (categorical: yes/no) | Questionnaire to mother | Q: Has anyone <u>ever</u> thought your child might have a problem with clumsiness, movement, or coordination? | Child aged 103 months |
| | Tympanostomy tubes fitted at any time (categorical: yes/no) | Questionnaires to mother and hearing assessment | Categorized as "yes" if the responses to any of the following was positive. Q: Has your child ever had tubes put in her ears? Has your child ever had surgery for tube insertion?<br><br>And observation of tubes during hearing assessment | Child aged 69 and 81 months (questionnaires); Focus @ 7 (hearing assessment) |
| | Hearing impairment (categorical: yes/no) | Pure tone audiometry | Categorized as "yes" if either ear had average air conduction thresholds at 1, 2, and 4 kHz, between 21 and 40 dB HL. Categorized as "no" if both ears had average air conduction thresholds at 1, 2, and 4 kHz better than or equal to 20 dB HL. | Focus @ 7 |

## References

Gathercole, S. E., & Baddeley, A. J. (1996). *The Children's Test of Nonword Repetition.* London, United Kingdom: The Psychological Corporation.

Goodyer, I., Wright, C., & Altham, P. M. E. (1989). Recent friendships in anxious and depressed school age children. *Psychological Medicine, 19*, 165–174.

Goodyer, I., Wright, C., & Altham, P. M. E. (1990). Recent achievements and adversities in anxious and depressed school age children. *Journal of Child Psychology & Psychiatry*, *31*, 1063–1077.

Manly, T., Robertson, I. H., Anderson, V., & Nimmo-Smith, I. (1998). *The Test of Everyday Attention for Children (TEA-Ch)*. Bury St Edmunds, United Kingdom: Thames Valley Test Company.

Northstone, K., Bonnell, S., Sadler, S., Carmichael, A., & the Focus@7 study team. (2005). *Avon Longitudinal Study of Parents and Children: Focus@7 built files* (No. v3c). Bristol, United Kingdom: ALSPAC.

Northstone, K., Bonnell, S., Horwood, J., Bell, C., Sadler, S., Carmichael, A., & the Focus@8 study team. (2006). *Avon Longitudinal Study of Parents and Children: Focus@8 built files documentation* (No. v3a). Bristol, United Kingdom: ALSPAC.

Rosner, J., & Simon, D. P. (1971). The Auditory Analysis Test: An initial report. *Journal of Learning Disabilities, 4,* 40–48.

Rust, J. (1996). *Wechsler Objective Language Dimensions manual.* London, United Kingdom: The Psychological Corporation.

Wechsler, D. (1992) *Wechsler Intelligence Scale for Children–Third UK Edition*. London, United Kingdom: The Psychological Corporation.

---

[i] 'O'level was the qualification obtained at age 16 when the parents of the cohort were at school.

[ii] "Focus" is the name of the assessment clinics that children from the Avon Longitudinal Study of Parents and Children (ALSPAC) sample were invited to attend. Focus@8 was the name of the clinic that children attended at age 8 years. Focus@7 was the name of the clinic they attended at age 7 years.
