# Supplementary material for: Prevalence and Predictors of Persistent Speech Sound Disorder at Eight Years Old: Findings From a Population Cohort Study
Source: J Speech Lang Hear Res. 2016 Aug;59(4):647–73. doi: 10.1044/2015_JSLHR-S-14-0282 (PMC5280061; doi:10.1044/2015_JSLHR-S-14-0282)
Supplement: Supplemental Table 3. [file JSLHR-59-647-s003.pdf]

Supplemental Table 3. Results of first stage univariable analysis and descriptive statistics for demographic and early childhood variables associated with persistent speech sound disorder (SSD; reporting all *p*s including those > .1, which were not carried through to the within-groups stage analysis). Bold values indicate *p* < .1.

**Grouped variables[a]**	**Category‡**	**Total sample *N***	**Case children summary data[b]**	**Rest of the cohort summary data[b]**	**Univariable model odds ratio [95% CI]**	***p***
**Demographics**						
Gender*	Female	6662	94 (2.8)	3303 (97.2)	1.0	**< .001**
	Male		169 (5.2)	3096 (94.8)	1.92 [1.48, 2.48]	
Ethnicity*	White	6066	233 (4.0)	5603 (96.0)	1.0	.69
	Non-White		8 (3.5)	222 (96.5)	0.87 [0.42, 1.78]	
Level of maternal education*	[i]O'level	6166	79 (3.6)	2093 (96.4)	1.0	**.025**
	< O'level		70 (5.2)	1265 (94.8)	1.47 [1.05, 2.04]	
	> O'level		93 (3.5)	2566 (96.5)	0.96 [0.71, 1.30]	
Maternal occupation*†	Nonmanual	5909	156 (3.3)	4616 (96.7)	1.0	**< .001**
	Manual		64 (5.6)	1073 (94.4)	1.76 [1.31, 2.38]	
Home ownership*	Mortgages/owned	6199	174 (3.4)	5007 (96.6)	1.0	**< .001**
	Rented/other		70 (6.9)	948 (93.1)	2.12 [1.60, 2.83]	
Maternal age**		6382	29.2 (4.8)	29.1 (4.6)	1.01 [0.98, 1.03]	.72
**Environment**						
Parity*	1st child	6161	84 (2.9)	2805 (97.1)	1.0	**< .001**
	2nd child		100 (4.6)	2068 (95.4)	1.61 [1.20, 2.17]	
	3rd child or more		55 (5.0)	1049 (95.0)	1.75 [1.24, 2.48]	
Languages other than English used in the home*	No	5399	194 (3.8)	4959 (96.2)	1.0	**.024**
	Yes		17 (6.9)	229 (93.1)	1.90 [1.14, 3.17]	
Child attends day nursery/crèche at age 8 weeks*	No	6067	232 (3.9)	5792 (96.1)	1.0	.79
	Yes		2 (4.7)	41 (95.3)	1.22 [0.29, 5.07]	
Child attends day nursery/crèche at age 8 months*	No	5874	219 (3.9)	5411 (96.1)	1.0	.40
	Yes		7 (2.9)	237 (97.1)	0.73 [0.34, 1.57]	
Child attends day nursery/crèche at age 15 months*	No	5888	211 (3.9)	5270 (96.1)	1.0	.93
	Yes		16 (3.9)	391 (96.1)	1.02 [0.61, 1.72]	
Child attends day nursery/crèche at age 24	No	5768	206 (4.0)	4965 (96.0)	1.0	.16

months*	Yes		17 (2.9)	580 (97.1)	0.71 [0.43, 1.17]	
Child attends playgroup, nursery, or a childminder at age 33 months*	No	2763	68 (4.0)	1656 (96.0)	1.0	.39
	Yes		48 (4.6)	991 (95.4)	1.18 [0.81, 1.72]	
Child attends day nursery/crèche at age 38 months*	No	5770	150 (4.2)	3441 (95.8)	1.0	.091
	Yes		72 (3.3)	2107 (96.7)	0.78 [0.59, 1.04]	
Child attends playgroup, nursery or childminder at age 47 months*	No	5583	13 (8.1)	148 (91.9)	1.0	**.013**
	Yes		203 (3.7)	5219 (96.3)	0.44 [0.25, 0.79]	
Child attends day nursery/crèche at age 54 months*	No	4756	17 (3.7)	440 (96.3)	1.0	.88
	Yes		166 (3.9)	4133 (96.1)	1.04 [0.63, 1.73]	
Mum reads to child at age 18 months*	Almost daily	5973	148 (3.4)	4219 (96.6)	1.0	**.003**
	3–5 times per week		52 (4.7)	1050 (95.3)	1.41 [1.02, 1.95]	
	< 3 times per week		32 (6.3)	472 (93.7)	1.93 [1.30, 2.87]	
Partner reads to child at age 18 months*	Almost daily	5750	64 (3.2)	1929 (96.8)	1.0	**.035**
	3–5 times per week		51 (3.5)	1412 (96.5)	1.09 [0.75, 1.58]	
	< 3 times per week		107 (4.7)	2187 (95.3)	1.47 [1.08, 2.02]	
Either parent reads to child at age 18 months*	Almost daily	5984	168 (3.5)	4612 (96.5)	1.0	**.012**
	3–5 times per week		44 (5.1)	814 (94.9)	1.48 [1.06, 2.09]	
	< 3 times per week		21 (6.1)	325 (93.9)	1.77 [1.11, 2.83]	
Mum reads to child at age 24 months*	Almost daily	5795	126 (3.4)	3593 (96.6)	1.0	**.013**
	3–5 times per week		66 (4.4)	1421 (95.6)	1.32 [0.98, 1.80]	
	< 3 times per week		34 (5.8)	555 (94.2)	1.75 [1.18, 2.58]	
Mum reads to child at age 42 months*	Almost daily	5727	140 (3.7)	3620 (96.3)	1.0	.45
	1–5 times per week		62 (4.5)	1314 (95.5)	1.22 [0.90, 1.66]	
	< Once per week		23 (3.9)	568 (96.1)	1.05 [0.67, 1.64]	
Partner reads to child at age 42 months*	Almost daily	5434	69 (4.2)	1590 (95.8)	1.0	.77
	1–5 times per week		61 (3.7)	1587 (96.3)	0.86 [0.62, 1.26]	
	< Once per week		81 (3.8)	2046 (96.2)	0.91 [0.66, 1.27]	
Another person reads to child at age 42 months*	Almost daily	2793	18 (3.9)	445 (96.1)	1.0	.68
	1–5 times per week		35 (4.1)	812 (95.9)	1.07 [0.60, 1.90]	
	< Once per week		51 (3.4)	1432 (96.6)	0.88 [0.51, 1.52]	
Overcrowding index at 8 weeks gestation*§	≤ 0.5	6132	90 (3.0)	2913 (97.0)	0.1	**< .001**
	> 0.5–0.75		80 (4.2)	1834 (95.8)	1.41 [1.04, 1.92]	
	> 0.75–1		45 (4.6)	929 (95.4)	1.57 [1.09, 2.26]	
	> 1		22 (9.1)	219 (90.9)	3.25 [2.00, 5.29]	

Overcrowding index at age 21 months*§	≤ 0.5	5522	37 (3.0)	1180 (97.0)	1.0	**.006**
	> 0.5–0.75		71 (3.2)	2152 (96.8)	1.05 [0.70, 1.58]	
	> 0.75–1		46 (5.0)	875 (95.0)	1.68 [1.08, 2.61]	
	> 1		59 (5.1)	1102 (94.9)	1.71 [1.12, 2.60]	
Overcrowding index at age 33 months*§	≤ 0.5	5501	40 (3.7)	1052 (96.3)	1.0	**.083**
	> 0.5–0.75		70 (3.2)	2108 (96.8)	0.87 [0.59, 1.30]	
	> 0.75–1		91 (4.8)	1817 (95.2)	1.32 [0.90, 1.93]	
	> 1		12 (3.7)	311 (96.3)	1.01 [0.53, 1.96]	
Family history of speech and language therapy attendance*	No	6135	218 (3.7)	5642 (96.3)	1.0	**.007**
	Yes		20 (7.3)	255 (92.7)	2.03 [1.26, 3.26]	
Premature birth*	No	6382	237 (3.9)	5773 (96.1)	1.0	.86
	Yes		14 (3.8)	358 (96.2)	0.95 [0.55, 1.65]	
Method of delivery*	Spontaneous	4434	152 (4.5)	3198 (95.5)	1.0	.11
	Assisted		21 (3.6)	563 (96.4)	0.78 [0.49, 1.25]	
	Elective caesarean		6 (3.2)	182 (96.8)	0.69 [0.30, 1.59]	
	Emergency caesarean		22 (7.1)	290 (92.9)	1.60 [1.00, 2.54]	
Pregnancy complications—Hypertension*	No	6662	256 (3.9)	6232 (96.1)	1.0	.96
	Yes		7 (4.0)	167 (96.0)	1.02 [0.47, 2.20]	
Pregnancy complications—Unexplained abdominal pain*	No	6662	247 (3.9)	6081 (96.1)	1.0	.43
	Yes		16 (4.8)	318 (95.2)	1.24 [0.74, 2.08]	
Pregnancy complications—Vaginal bleeding*	No	6662	231 (3.8)	5822 (96.2)	1.0	**.096**
	Yes		32 (5.3)	577 (94.8)	1.40 [0.96, 2.04]	
Pregnancy complications—Vomiting*	No	6662	233 (3.9)	5710 (96.1)	1.0	.75
	Yes		30 (4.2)	689 (95.8)	1.07 [0.72, 1.57]	
Any pregnancy complications*	No	6662	189 (3.9)	4672 (96.1)	1.0	.68
	Yes		74 (4.1)	1727 (96.9)	1.06 [0.81, 1.39]	
Child was breastfed*	Never	5896	48 (4.5)	1029 (95.5)	1.0	.51
	For less than 3 months		76 (4.0)	1805 (96.0)	0.90 [0.62, 1.31]	
	For 3 or more months		108 (3.7)	2830 (96.3)	0.82 [0.58, 1.16]	
Ever smoked*	No	6224	124 (3.6)	3310 (96.4)	1.0	.21

	Yes		118 (4.2)	2672 (95.8)	1.18 [0.91, 1.52]	
Smoked pre-pregnancy*	No	6255	171 (3.7)	4482 (96.3)	1.0	.15
	Yes		72 (4.5)	1530 (95.5)	1.23 [0.93, 1.63]	
Smoked in 1st trimester*	No	6255	190 (3.7)	4937 (96.3)	1.0	.13
	Yes		53 (4.7)	1075 (95.3)	1.28 [0.94, 1.75]	
Smoked during last 2 weeks*	No	6255	196 (3.6)	5189 (96.4)	1.0	**.017**
	Yes		47 (5.4)	823 (94.6)	1.51 [1.09, 2.10]	
Mother had her first child as a teenager*	No	6251	209 (3.8)	5262 (96.2)	1.0	.58
	Yes		33 (4.2)	747 (95.8)	1.11[0.76, 1.62]	
Depression subscale of the Edinburgh Post Natal Depression Scales (EPDS) at 18 weeks gestation**		5889	6.6 (4.4)	6.5 (4.6)	1.00 [0.98, 1.03]	.78
Depression subscale of the EPDS at 32 weeks gestation**		6045	6.5 (4.5)	6.7 (4.8)	0.99 [0.97, 1.02]	.64
Depression subscale of the EPDS at age 8 weeks**		6063	6.0 (4.7)	5.8 (4.5)	1.01 [0.98, 1.04]	.43
Depression subscale of the EPDS at age 8 months**		5979	5.0 (4.3)	5.2 (4.5)	0.99 [0.96, 1.02]	.52
Depression subscale of the EPDS at age 21 months**		5766	5.4 (4.6)	5.5 (4.6)	1.00 [0.97, 1.03]	.80
Depression subscale of the EPDS at age 33 months**		5597	5.9 (4.8)	6.1 (4.9)	0.99 [0.96, 1.02]	.54
Anxiety subscale of the Crown–Crisp Experiential Index (CCEI) at 18 weeks gestation**		5840	4.5 (3.5)	4.7 (3.4)	0.98 [0.94, 1.02]	.43
Depression subscale of the CCEI at 18 weeks gestation**		5840	4.0 (2.7)	4.2 (2.9)	0.97 [0.93, 1.02]	.25
Somatic subscale of the CCEI at 18 weeks gestation**		5840	4.1 (2.3)	4.1 (2.3)	0.99 [0.93, 1.05]	.71
Total of subscales of the CCEI at 18 weeks gestation**		5840	12.6 (7.1)	13.0 (7.3)	0.99 [0.97, 1.01]	.34
Anxiety subscale of the CCEI at 32 weeks gestation**		5982	4.8 (3.5)	4.9 (3.5)	0.99 [0.95, 1.03]	.69
Depression subscale of the CCEI at 32 weeks gestation**		5982	4.7 (3.1)	4.8 (3.1)	0.99 [0.95, 1.03]	.70
Somatic subscale of the CCEI at 32 weeks		5982	5.1 (2.9)	5.0 (2.6)	1.01 [0.96, 1.07]	.59

gestation**						
Total of subscales of the CCEI at 32 weeks gestation**		5982	14.6 (8.0)	14.6 (7.9)	1.00 [0.98, 1.02]	.88
Anxiety subscale of the CCEI at age 8 weeks**		6061	3.3 (3.2)	3.3 (3.1)	1.00 [0.96, 1.04]	.97
Depression subscale of the CCEI at age 8 weeks**		6061	3.5 (2.9)	3.4 (2.9)	1.02 [0.97, 1.06]	.51
Somatic subscale of the CCEI at age 8 weeks**		6061	2.6 (1.7)	2.5 (1.6)	1.03 [0.95, 1.11]	.51
Total of subscales of the CCEI at age 8 weeks**		6061	9.3 (6.6)	9.1 (6.6)	1.00 [0.99, 1.02]	.64
Anxiety subscale of the CCEI at age 8 months**		5978	3.4 (3.2)	3.5 (3.2)	0.99 [0.95, 1.04]	.76
Depression subscale of the CCEI at age 8 months**		5978	3.3 (2.8)	3.2 (2.9)	1.01 [0.97, 1.06]	.53
Somatic subscale of the CCEI at age 8 months**		5978	2.5 (1.8)	2.5 (1.7)	1.00 [0.93, 1.08]	.97
Total of subscales of the CCEI at age 8 months**		5978	9.3 (6.6)	9.2 (6.8)	1.00 [0.98, 1.02]	.90
Anxiety subscale of the CCEI at age 21 months**		5767	3.4 (3.1)	3.7 (3.2)	0.97 [0.93, 1.02]	.21
Depression subscale of the CCEI at age 21 months**		5766	2.9 (2.7)	2.8 (2.6)	1.01 [0.96, 1.06]	.75
Somatic subscale of the CCEI at age 21 months**		5765	2.6 (2.1)	2.6 (1.8)	1.01 [0.94, 1.08]	.85
Total of subscales of the CCEI at age 21 months**		5767	9.6 (7.2)	9.7 (6.9)	1.00 [0.98, 1.02]	.78
Anxiety subscale of the CCEI at age 33 months**		5603	4.6 (3.6)	4.6 (3.4)	0.99 [0.96, 1.03]	.77
Depression subscale of the CCEI at age 33 months**		5595	4.0 (3.0)	4.1 (3.1)	0.99 [0.95, 1.04]	.73
Somatic subscale of the CCEI at age 33 months**		5602	2.8 (1.9)	2.8 (2.0)	1.00 [0.94, 1.08]	.90
Total of subscales of the CCEI at age 33 months**		5601	11.4 (7.4)	11.5 (7.4)	1.00 [0.98, 1.02]	.81
**Early speech and language performance**						
MacArthur–Bates Understanding Vocabulary score at 38 months**		5770	10.3 (2.6)	11.1 (2.3)	0.91 [0.87, 0.95]	< **.001**
MacArthur–Bates Saying Vocabulary score at 38 months**		5770	9.9 (3.2)	11.4 (1.6)	0.77 [0.73, 0.80]	< **.001**
Intelligibility to mother at 38 months*	Mostly	5714	197 (3.5)	5378 (96.5)	0.1	< **.001**
	Sometimes/rarely		23 (16.5)	116 (83.5)	5.41 [3.39, 8.66]	
Intelligibility to family at 38 months*	Mostly	5712	150 (2.9)	5051 (97.1)	0.1	< **.001**

	Sometimes/rarely		68 (13.3)	443 (86.7)	5.17 [3.82, 6.99]	
Intelligibility to others at 38 months*	Mostly	5703	111 (2.4)	4526 (97.6)	0.1	< **.001**
	Sometimes/rarely		107 (10.0)	959 (90.0)	4.55 [3.46, 5.99]	
Uses gestures at or before 38 months*	No, never did	5696	37 (2.2)	1632 (97.8)	1.0	< **.001**
	Yes, but not now		127 (3.5)	3465 (96.5)	1.62 [1.12, 2.34]	
	Yes, still does		57 (13.1)	378 (86.9)	6.65 [4.33, 10.21]	
Word combination at 24 months *	Often	5628	66 (2.0)	3171 (98.0)	1.0	< **.001**
	Sometimes		74 (4.8)	1462 (95.2)	2.43 [1.74, 3.41]	
	Not yet		72 (8.4)	783 (91.6)	4.42 [3.14, 6.23]	
Word combination at 38 months*	Often	5641	174 (3.3)	5121 (96.7)	1.0	< **.001**
	Sometimes		25 (8.7)	262 (91.3)	2.81 [1.81, 4.35]	
	Not yet		18 (30.5)	41 (69.5)	12.92 [7.28, 22.95]	
Word morphology at 38 months**		5711	7.6 (3.5)	9.5 (2.8)	0.84 [0.81, 0.88]	< **.001**
Irregular grammar at 24 months**		5750	13.1 (12.4)	19.5 (13.8)	0.96 [0.95, 0.97]	< **.001**
Stuttering*	Never	5675	111 (4.1)	2606 (95.9)	1.0	.45
	Sometimes		91 (3.5)	2549 (96.6)	0.84 [0.63, 1.11]	
	Often		13 (4.1)	305 (95.9)	1.00 [0.56, 1.80]	
Denver Communication Scale at 6 months**		5561	–0.03 (1.01)	–0.02 (0.96)	1.00 [0.87, 1.14]	.95
Denver Communication Scale at 18 months**		5775	–0.33 (1.10)	0.04 (0.98)	0.68 [0.59, 0.78]	< **.001**
**Early literacy and learning**						
School entry assessment (age 4–5 years): Reading*	Achieved level	4633	54 (7.2)	698 (92.8)	1.0	< **.001**
	Above expectations		130 (3.4)	3751 (96.7)	0.45 [0.32, 0.62]	
School entry assessment (age 4–5 years): Writing*	Achieved level	4634	76 (6.1)	1178 (93.9)	1.0	< **.001**
	Above expectations		108 (3.2)	3272 (96.8)	0.51 [0.38, 0.69]	
**Other early developmental**						
Low birth weight*	> 2500 grams	6308	232 (3.9)	5746 (96.1)	1.0	.55
	≤ 2500 grams		15 (4.6)	315 (95.5)	1.18 [0.69, 2.01]	
Avon Longitudinal Study of Parents and Children (ALSPAC) Developmental Scale Social score at 6 months**		5558	–0.06 (1.07)	–0.00 (0.99)	0.94 [0.82, 1.08]	.36
ALSPAC Developmental Scale Fine Motor at 6 months**		5537	–0.18 [1.05)	–0.00 (0.98)	0.83 [0.73, 0.96]	**.009**
ALSPAC Developmental Scale Gross Motor at 6		5547	–0.04 (0.94)	0.01 (0.97)	0.95 [0.83, 1.09]	.48

months**						
ALSPAC Developmental Scale Social score at 18 months**		5786	–0.17 (1.11)	0.01 (0.98)	0.83 [0.72, 0.95]	**.005**
ALSPAC Developmental Scale Fine Motor score at 18 months**		5757	–0.11 (1.11)	0.05 (0.96)	0.86 [0.75, 0.98]	**.023**
ALSPAC Developmental Scale Gross Motor at 18 months**		5783	–0.22 (1.29)	–0.00 (0.94)	0.82 [0.73, 0.92]	**.002**
ALSPAC Developmental Scale Social score at 30 months**		5142	–0.26 (1.05)	0.02 (0.98)	0.76 [0.66, 0.87]	**< .001**
ALSPAC Developmental Scale Fine Motor score at 30 months**		5121	–0.14 (1.06)	0.06 (0.97)	0.82 [0.72, 0.94]	**.004**
ALSPAC Developmental Scale Gross Motor score at 30 months**		5132	–0.32 (1.26)	–0.01 (0.96)	0.76 [0.67, 0.86]	**< .001**
ALSPAC Developmental Scale Social score at 42 months**		5328	–0.24 (1.22)	0.04 (0.95)	0.76 [0.67, 0.87]	**< .001**
ALSPAC Developmental Scale Fine Motor score at 42 months**		5332	–0.32 (1.13)	0.06 (0.97)	0.71 [0.63, 0.80]	**< .001**
ALSPAC Developmental Scale Gross Motor score at 42 months**		5335	–0.41 (1.26)	0.01 (0.96)	0.70 [0.62, 0.78]	**< .001**
School entry assessment (age 4–5 years): Large motor*	Achieved level	1549	27 (7.9)	313 (92.1)	1.0	**< .001**
	Above expectations		40 (3.3)	1169 (96.7)	0.40 [0.24, 0.66]	
School entry assessment (age 4–5 years): Fine motor*	Achieved level	1739	16 (4.1)	372 (95.9)	1.0	.79
	Above expectations		60 (4.4)	1291 (95.6)	1.08 [0.62, 1.90]	
Laterality at 42 months*	Right	5700	138 (3.6)	3665 (96.4)	1.0	**.069**
	Mixed/left		88 (4.6)	1809 (95.4)	1.29 [0.98, 1.70]	
Weak sucking at 4 weeks*	No	6158	179 (3.5)	4879 (96.5)	1.0	**.009**
	Yes		58 (5.3)	1042 (94.7)	1.52 [1.12, 2.06]	
Choking at 4 weeks*	No	6158	108 (4.1)	2511 (95.9)	1.0	.34
	Yes		129 (3.7)	3410 (96.4)	0.88 [0.68, 1.1]	
Dribbling at 4 weeks*	No	6158	107 (4.7)	2194 (95.4)	1.0	**.013**
	Yes		130 (3.4)	3727 (96.6)	0.72 [0.55, 0.93]	
Drinking too fast at 4 weeks*	No	6158	49 (5.0)	930 (95.0)	1.0	**.048**
	Yes		188 (3.6)	4991 (96.4)	0.71 [0.52, 0.99]	
Exhaustion with feeding at 4 weeks*	No	6158	121 (3.8)	3059 (96.2)	1.0	.85
	Yes		116 (3.9)	2862 (96.1)	1.02 [0.79, 1.33]	
Slow feeding at 4 weeks*	No	6158	141 (3.8)	3545 (96.2)	1.0	.91

	Yes		96 (3.9)	2376 (96.1)	1.02 [0.78, 1.32]	
Small quantity feeds at 4 weeks*	No	6158	120 (3.9)	2944 (96.1)	1.0	.78
	Yes		117 (3.8)	2977 (96.2)	0.96 [0.74, 1.25]	
Baby being unsatisfied*	No	6158	106 (3.7)	2739 (96.3)	1.0	.64
	Yes		131 (4.0)	3182 (96.0)	1.06 [0.82, 1.38]	
Refusing milk*	No	6158	206 (3.7)	5295 (96.3)	1.0	.23
	Yes		31 (4.7)	626 (95.3)	1.27 [0.86, 1.87]	
Difficulties feeding*	No	6127	195 (3.6)	5184 (96.4)	1.0	**.088**
	Yes		37 (5.0)	711 (95.1)	1.38 [0.97, 1.98]	

[a]This column shows how the variables were grouped in the second stage within group multivariable analysis. [b]Where the variable of interest is categorical, the two numbers refer to *n* and (%) where % is the percentage within the factor category. The reference category for each variable can be identified by its odds ratio of 1.0. Where the variable of interest is continuous, the numbers are mean (and standard deviation) and the odds ratio relates to the change in odds for a one-unit increase in the exposure variable. The exception to this is the odds ratio for IQ and the MacArthur–Bates scores, which are based on a change of ten units.
[*]Categorical. [**]Continuous. [‡]For categorical variables only. [†]Supplemented with father’s social class when the mother’s was not available. [§]People per room.

---

[i]O’level was the qualification obtained at age 16 years when the parents of the cohort were at school.
