# Supplementary material for: Prevalence and Predictors of Persistent Speech Sound Disorder at Eight Years Old: Findings From a Population Cohort Study
Source: J Speech Lang Hear Res. 2016 Aug;59(4):647–73. doi: 10.1044/2015_JSLHR-S-14-0282 (PMC5280061; doi:10.1044/2015_JSLHR-S-14-0282)
Supplement: Supplemental Table 4. [file JSLHR-59-647-s004.pdf]

**Supplemental Table 4.** Results of first stage univariable analysis and descriptive statistics for demographic and school-age predictor variables associated with persistent speech sound disorder (reporting all *p*s including those > .1, which were not carried through to the within-groups stage analysis). Bold values indicate p < .1.

| **Grouped variables[a]** | **Specific variables** | **Data available for each variable: *N*** | **Total sample *N*** | **Case children summary data[b]** | **Rest of the cohort summary data[b]** | **Univariable model odds ratio [95% CI]** | ***p*** |
|---|---|---|---|---|---|---|---|
| **Demographics** | | | | | | | |
| Gender * | Female | 3397 | 6662 | 94 (2.8) | 3303 (97.2) | 1.0 | **< .001** |
| | Male | 3265 | | 169 (5.2) | 3096 (94.8) | 1.92 [1.48, 2.48] | |
| Ethnicity * | White | 5836 | 6066 | 233 (3.9) | 5603 (96.0) | 1.0 | .69 |
| | Non-White | 230 | | 8 (3.4) | 222 (96.5) | 0.87 [0.42, 1.78] | |
| Level of maternal education * | [i]O’level | 2172 | 6166 | 79 (3.6) | 2093 (96.4) | 1.0 | **.025** |
| | < O’level | 1335 | | 70 (5.2) | 1265 (94.8) | 1.47 [1.05, 2.04] | |
| | > O’level | 2659 | | 93 (3.5) | 2566 (96.5) | 0.96 [0.71, 1.30] | |
| Maternal occupation * | Non-manual | 4772 | 5909 | 156 (3.3) | 4616 (96.7) | 1.0 | **< .001** |
| | Manual | 1137 | | 64 (5.6) | 1073 (94.4) | 1.76 [1.31, 2.38] | |
| Home ownership * | Mortgages/owned | 5181 | 6199 | 174 (3.4) | 5007 (96.6) | 1.0 | **< .001** |
| | Rented/other | 1018 | | 70 (6.9) | 948 (93.1) | 2.12 [1.60, 2.83] | |
| Maternal age ** | | 6382 | 6382 | 29.2 (4.8) | 29.1 (4.6) | 1.01 [0.98, 1.03] | .72 |
| | | | | | | | |
| **Concurrent speech and language performance** | | | | | | | |
| Language comprehension** | | 6655 | 6655 | 7.2 (2.3) | 7.4 (1.9) | 0.95 [0.89, 1.01] | **.094** |
| Diadochokinetic (DDK) tasks: *pe** | Correct | 4333 | 6589 | 159 (3.7) | 4174 (96.3) | 1.0 | |
| | Incorrect | 2286 | 6655 | 100 (4.4) | 2186 (95.6) | 1.20 [0.93, 1.55] | .16 |
| DDK tasks: *te** | Correct | 5617 | 6617 | 209 (3.7) | 5408 (96.3) | 1.0 | |
| | Incorrect | 1002 | | 49 (4.9) | 953 (95.1) | 1.33 [0.97, 1.83] | **.088** |
| DDK tasks: *ke** | Correct | 1590 | 6618 | 52 (3.3) | 1538 (96.7) | 1.0 | |
| | Incorrect | 5028 | | 207 (4.1) | 4821 (95.9) | 1.27 [0.93,1.73] | .12 |
| DDK tasks: *peteke** | Correct | 1581 | 6616 | 47 (3.0) | 1534 (97.0) | 1.0 | |
| | Incorrect | 5035 | | 212 (4.2) | 4823 (95.8) | 1.43 [1.04, 1.98] | **.023** |
| DDK tasks: *bedege** | Correct | 973 | 6619 | 26 (2.7) | 947 (97.3) | 1.0 | |
| | Incorrect | 5646 | | 233 (4.1) | 5413 (95.9) | 1.57 [1.04, 2.37] | **.023** |
| Phoneme deletion** | | 5998 | 5998 | 16.0 (10.1) | 20.5 (9.3) | 0.95 [0.94, 0.96] | **< .001** |

| | | | | | | | |
|---|---|---|---|---|---|---|---|
| Difficulty pronouncing sounds* | No | 4773 | 5432 | 111 (2.3) | 4662 (97.7) | 1.0 | < **.001** |
| | Yes | 659 | | 95 (14.4) | 564 (85.6) | 7.07 [5.31, 9.43] | |
| Nonword repetition** | | 6640 | 6640 | 5.7 (3.0) | 7.3 (2.5) | 0.78 [0.75, 0.82] | < **.001** |
| | | | | | | | |
| **Concurrent literacy and learning** | | | | | | | |
| Reading test** | | 6006 | 6006 | 23.8 (10.7) | 28.8 (9.0) | 0.94 [0.93, 0.96] | < **.001** |
| Spelling test** | | 5945 | 5945 | 20.9 (13.7) | 26.3 (12.5) | 0.97 [0.96, 0.98] | < **.001** |
| School assessment: Reading* | Achieved expected level | 2842 | 5644 | 111 (3.9) | 2731 (96.1) | 1.0 | < **.001** |
| | Underachieved | 513 | | 50 (9.8) | 463 (90.3) | 2.66 [1.88, 3.76] | |
| | Exceeded expected level | 2289 | | 65 (2.8) | 2224 (97.2) | 0.72 [0.53, 0.98] | |
| School assessment: Writing* | Achieved expected score | 4502 | 5640 | 153 (3.4) | 4349 (96.6) | 1.0 | < **.001** |
| | Underachieved | 536 | | 60 (11.2) | 476 (88.8) | 3.58 [2.62, 4.90] | |
| | Exceeded expected level | 602 | | 13 (2.3) | 589 (97.8) | 0.63 [0.35, 1.11] | |
| Identified learning problems* | No | 4948 | 5434 | 162 (3.3) | 4786 (96.7) | 1.0 | < **.001** |
| | Yes | 486 | | 50 (10.3) | 436 (89.7) | 3.39 [2.43, 4.72] | |
| | | | | | | | |
| **Other developmental (concurrent)** | | | | | | | |
| Verbal IQ ** | | 6576 | 6576 | 10.1 (1.9) | 10.7 (1.7) | 0.80 [0.74, 0.86] | < **.001** |
| Performance IQ ** | | 6567 | 6567 | 9.4 (1.9) | 10.0 (1.7) | 0.81 [0.76, 0.88] | < **.001** |
| Combined IQ score ** | | 6548 | 6548 | 9.8 (1.9) | 10.4 (1.6) | 0.78 [0.72, 0.84] | < **.001** |
| Auditory memory ** | | 6425 | 6425 | 9.2 (3.4) | 10.4 (3.1) | 0.88 [0.84, 0.92] | < **.001** |
| Spatial ability ** | | 6560 | 6560 | 10.1 (4.3) | 10.6 (3.8) | 0.96 [0.93, 1.00] | **.030** |
| Attention ** | | 6390 | 6390 | 5.6 (2.4) | 5.2 (1.9) | 1.08 [1.03, 1.13] | **.004** |
| Friendships ** | | 6360 | 6360 | 3.8 (2.8) | 3.4 (2.4) | 1.05 [1.00, 1.11] | **.037** |
| Suspected coordination problem * | No | 5400 | 5710 | 184 (3.4) | 5216 (96.6) | 1.0 | < **.001** |
| | Yes | 310 | 6360 | 32 (10.3) | 278 (89.7) | 3.26 [2.20, 4.84] | |
| Tympanostomy tubes fitted at any time * | No | 6020 | 6404 | 208 (3.5) | 5812 (96.5) | 1.0 | |
| | Yes | 384 | | 41 (10.7) | 343 (89.3) | 3.34 [2.35, 4.75] | < **.001** |
| Hearing impairment * | No | 5235 | 5666 | 178(3.4) | 5057 (96.6) | 1.0 | **.003** |
| | Yes | 431 | | 28 (6.5) | 403 (93.5) | 1.97 [1.31, 2.98] | |

[a]This column shows how the variables were grouped in the second stage within group multivariable analysis. [b]Where the variable of interest is categorical, the two numbers refer to n and (%) where % is the percentage within that factor. The reference category for each variable can be identified by its odds ratio of 1.0. Where the variable of interest is continuous, the numbers are mean (and standard deviation) and the odds ratio relates to the change in odds for a one-unit increase in the exposure variable. The exception to this is the odds ratio for IQ which is based on a change of ten units.

[*]Categorical variable. **Continuous variable.

[i] O'level was the qualification obtained at age 16 years when the parents of the cohort were at school.
